# Suspected and Non-Targeted Screening of Non-Edible Substances in Food by UPLC-Q-TOF-MS

**DOI:** 10.3390/foods15112001

**Published:** 2026-06-03

**Authors:** Ting Wang, Fuhong Chen, Lirong Pan, Wenxuan Yuan, Jie Pang, Xianliang Li, Cunxian Xi, Dunming Xu

**Affiliations:** 1 Technical Center of Xiamen Customs, Xiamen 361026, China; 15293594819@163.com (T.W.); chenfuhong1122@126.com (F.C.); 13400720050@163.com (L.P.); yuanwx1995@163.com (W.Y.); 2 College of Food Science, Fujian Agriculture and Forestry University, Fuzhou 350002, China; pang3721941@163.com; 3 Technical Center of Chongqing Customs, Chongqing 401147, China; cqciqlxl@163.com (X.L.); cqxicx@163.com (C.X.)

**Keywords:** non-edible substances, suspected screening, dispersive solid-phase extraction, unknown structural analogues, PDE-5 inhibitors, high-resolution mass spectrometry

## Abstract

A screening method based on dispersive solid-phase extraction (DSPE) coupled with ultra-performance liquid chromatography–quadrupole-time-of-flight mass spectrometry (UPLC-Q-TOF-MS) was established for the analysis of non-edible substances in food. This method is applicable to a wide range of non-edible substances, including but not limited to antihypertensive, hypoglycemic, weight-loss, antimicrobial, antipyretic–analgesic, sedative–hypnotic, and antifatigue agents. Through systematic optimization of sample pretreatment and UPLC-Q-TOF-MS conditions, ultrasonic extraction with methanol followed by cleanup using 25 mg Primary Secondary Amine (PSA) and 50 mg C_18_ was identified as the optimal procedure. The methodological validation demonstrated that all 38 quality control compounds exhibited excellent linear correlation coefficients (R^2^ > 0.99) across a concentration range of 0.005~5.0 mg/kg. At three spiking levels, the mean recoveries and relative standard deviations (RSDs) in four matrices ranged from 67.79% to 110.93% and from 0.23% to 9.37%, respectively. The screening detection limits (SDLs) and limits of quantification (LOQs) were within the range of 0.003~0.5 mg/kg. A screening database comprising 390 substances was constructed. In addition, an identification strategy for the unknown structural analogues was established by summarizing the mass spectrometric fragmentation patterns of the phosphodiesterase-5 (PDE-5) inhibitor analogues. Applied to 110 batches of samples, the method screened 12 known non-edible substances and identified a new PDE-5 inhibitor analogue, phenyl 3-desethyl 3-propyl carbodenafil. The workflow integrates suspected screening using a comprehensive database with a non-targeted identification strategy for unknown analogues. Overall, this strategy is efficient, sensitive and accurate, providing a robust analytical platform for high-throughput screening and discovery of illegally added unknown substances in food.

## 1. Introduction

With rapid socio-economic development and changes in lifestyle, public awareness of health has increased substantially, leading to a growing demand for functional food that extends beyond traditional health supplements to ordinary food products. However, in pursuit of exaggerated efficacy and market competitiveness, some unscrupulous manufacturers and distributors illegally add non-edible substances to food products. Non-edible substances are defined as chemical compounds that are not included in the national food safety standard approval list, lack any nutritional function or processing necessity, and are illegally added to food products. Moreover, these substances do not fall under the category of “dual-use food and medicinal substances” as designated by the National Health Commission of China, nor are they approved as ordinary food ingredients. They are also not listed in the National Food Safety Standard for Uses of Food Additives (GB 2760–2014), the relevant announcements on food additives issued by the National Health Commission, or the categories of nutrient fortifiers specified in the National Food Safety Standard for Uses of Food Nutrient Fortifiers (GB 14880–2012), nor do they comply with any other applicable laws and regulations [1]. Currently, illegally added non-edible substances can be generally classified into two major categories. The first category comprises industrial chemicals, such as melamine, malachite green, and Sudan Red, which are used to falsify sensory attributes or quality indicators for illicit economic gain [2,3]. The second category includes pharmacologically active substances, such as ephedrine, diazepam, and sildenafil, which are deliberately incorporated into functional foods to mislead consumers by enhancing their apparent physiological effects [4,5].

To safeguard food safety, regulatory authorities and relevant industries commit significant resources each year to systematic risk surveillance. In China, the current monitoring framework relies on a series of supplementary food testing standards issued by the State Administration for Market Regulation, for example, BJS201901 [6] and BJS201805 [7], which serve as the core technical guidelines [8,9]. These analytical methods are predominantly based on liquid chromatography–triple-quadrupole mass spectrometry (LC-MS/MS) operated in multiple-reaction monitoring (MRM) mode. By monitoring specific precursor-to-product ion transitions, MRM-based methods provide excellent selectivity and sensitivity in complex food matrices, enabling reliable qualitative and quantitative analysis [10,11].

Nevertheless, the high selectivity of LC-MS/MS also represents an inherent limitation when addressing unknown or emerging risks. Method development relies heavily on reference material, typically allowing only a limited and predefined set of target compounds—ranging from dozens to several hundred—to be monitored. Moreover, each MRM transition requires a finite dwell time; consequently, expanding the target list prolongs the overall scan cycle and results in an insufficient number of data points across chromatographic peaks, thereby adversely affecting quantitative accuracy and reproducibility [12]. As a result, conventional methods exhibit a distinct “regulatory gap” in response to the continual emergence of novel non-edible substances, limiting their capacity for comprehensive non-targeted or high-throughput screening.

To address these limitations, high-resolution mass spectrometry (HRMS), characterized by ultra-high resolving power, accurate mass measurement, and high sensitivity, has attracted increasing attention for the analysis of complex food matrices [13]. HRMS not only effectively discriminates against matrix interference but also enables the screening and identification of trace-level components, thereby offering both qualitative and quantitative analysis [14,15]. In the context of HRMS-based workflows, analytical strategies can be broadly categorized into targeted, suspected, and non-targeted screening. Targeted screening involves the quantification of predefined compounds using reference standards. Suspected screening relies on precursor exact mass error and MS/MS spectral libraries to identify compounds from a suspect list without necessarily having the standards at the time of screening. True non-targeted screening aims to identify completely unknown compounds without any prior information, often through sophisticated data analysis tools. For instance, Kapsi et al. developed a combined HPLC-HRMS and ICP-MS approach for the targeted and non-targeted detection of arsenolipids in tuna fish reference material (BCR-627), successfully confirming 11 known arsenolipids and revealing 26 unknown analogues [16]. Similarly, Huang et al. applied electron-activated dissociation (EDA) technology to analyze 181 synthetic cannabinoids and proposed an intelligent parsing algorithm, enabling non-targeted screening and automated structural elucidation of new psychoactive substances based on diagnostic fragment ions [17]. Furthermore, Gao et al. established a qualitative screening method for mycotoxins in maize using HPLC-HRMS with data-dependent acquisition. They developed a quantitative structure–retention relationship (QSRR) prediction model using machine learning to improve the confidence of suspect and non-targeted screening, while systematically optimizing key parameters and introducing a multi-metric evaluation system (accuracy, F1 score, MCC) to assess method performance [18].

Current research has largely focused on the development of screening databases for individual or limited classes of known drugs, whereas integrated approaches that combine database-driven broad-spectrum screening with systematic analysis of mass spectrometric fragmentation patterns to establish identification strategies for unknown structural analogues remain scarce. In this study, we developed a comprehensive workflow that combines suspected screening against a multi-category database of 390 non-edible substances with a non-targeted identification strategy for unknown structural analogues based on fragmentation pattern analysis.

Within the UPLC-Q-TOF-MS platform, information-dependent acquisition (IDA) mode triggers MS/MS fragmentation of qualified precursor ions during primary full-scan MS, allowing the simultaneous acquisition of extract ion chromatogram (XIC) data, precursor exact-mass (MS) information, isotope pattern and fragment ion (MS/MS) spectra from a single injection. This multidimensional data acquisition substantially enhances the confidence of compound identification and the characterization of unknown substances [19]. Data acquired via IDA enable rapid and high-confidence library matching of suspected compounds by integrating accurate mass measurements with MS/MS fragmentation information. For unknown compounds, the precursor exact mass, together with its isotope pattern and MS/MS fragmentation data, can be collectively analyzed to infer the elemental composition and plausible structural features, thereby facilitating effective non-targeted identification [20].

Furthermore, effective sample pretreatment is essential for removing interferences and minimizing instrument contamination. While the traditional dilute-and-shoot approach is operationally simple, it introduces substantial matrix components into the analytical system, potentially compromising long-term performance [21]. Conventional solid-phase extraction offers satisfactory cleanup but is often hampered by cumbersome procedures, intensity of retention time, and high solvent consumption, rendering it less suitable for high-throughput screening demands. Dispersive solid-phase extraction (DSPE) has emerged as an advanced sample pretreatment technique derived from traditional solid-phase extraction. In DSPE, the adsorbent is directly dispersed into the sample extract after organic solvent extraction. Through vigorous mixing such as vortexing or shaking, the adsorbent achieves intimate contact with the sample matrix, allowing efficient adsorption of various interfering substances. The adsorbent is subsequently separated from the extract by centrifugation. Recent studies have demonstrated the successful application of DSPE combined with LC-MS/MS for the determination of various substances in complex food matrices. For instance, Li et al. developed a DSPE-UPLC-MS/MS method for the determination of six diuretic stimulants in health tea using a combination of C_18_ and HLB as dispersive sorbents [22]. Niu et al. synthesized a novel metal–organic framework-based molecularly imprinted polymer (UiO-66-NH_2_@MIPs) and applied it as a selective DSPE adsorbent for the enrichment of fluoroquinolones in aquatic products prior to LC-MS/MS analysis [23]. These studies highlight the versatility and effectiveness of DSPE as a sample pretreatment technique for food safety monitoring. DSPE has received significant attention due to its notable advantages in extraction efficiency, reduced solvent usage, operational simplicity, and broad applicability across diverse food matrices, and has been successfully implemented in food testing applications [24,25,26].

In this study, 38 representative non-edible substances with diverse physicochemical properties were selected as quality control compounds. The DSPE technique was integrated with UPLC-Q-TOF-MS operated in IDA mode, and chromatographic, mass spectrometric, and sample pretreatment conditions were systematically optimized. On this basis, a comprehensive screening database comprising 390 non-edible substances was established, encompassing multiple categories such as antihypertensive, hypoglycemic, weight-loss, antimicrobial, antipyretic–analgesic, sedative–hypnotic, and antifatigue compounds. The established method is simple to operate and rapid in analysis, enabling the screening of illegally added non-edible substances in food with a single injection and without the need for reference standards, thereby rendering it suitable for high-throughput analysis of large sample sets. Furthermore, leveraging the rich MS/MS information acquired under IDA mode, the mass spectrometric fragmentation patterns of PDE-5 inhibitor analogues were systematically summarized, and a strategy was developed for the identification and confirmation of unknown structural analogues in samples. Finally, the method was applied to 110 commercially available food samples, resulting in the successful screening and identification of 13 known and unknown non-edible substances. These results demonstrate the practical applicability of the proposed method for both suspected screening and non-targeted identification.

## 2. Materials and Methods

### 2.1. Instruments

An X500R QTOF mass spectrometer and Exion LC system were provided by SCIEX Co., Ltd., Framingham, MA, USA. An ultrapure water system was purchased from Jingyi Xingye Technology Co., Ltd., Beijing, China. A JY5002 electronic balance was purchased from Shanghai Sanheng Scientific Instrument Co., Ltd., Shanghai, China. An XS105DU electronic balance was purchased from Mettler Toledo, Greifensee, Switzerland. Sigma2-16PK and Sigma2-15PK centrifuges were purchased from Shanghai Bomaixing Instrument Co., Ltd., Shanghai, China. The MS200 multi-tube vortex mixer was purchased from Hangzhou Ruicheng Instrument Co., Ltd., Hangzhou, China. The MTV-100 vortex mixer was purchased from Hangzhou Aosheng Instrument Co., Ltd., Hangzhou, China. The KQ-700DE CNC ultrasonic cleaner was purchased from Kunshan Ultrasonic Instrument Co., Ltd., Kunshan, China.

### 2.2. Reagents and Materials

A Kinetex F5 chromatographic column (100 × 3.0 mm, 2.6 μm) was purchased from Phenomenex, Torrance, CA, USA. Nylon syringe filters (0.22 μm) were obtained from Beijing Naou Technology Co., Ltd., Beijing, China, and 1 mL sterile syringes were purchased from Jiangsu Kangyou Medical Equipment Co., Ltd., Taizhou, China. Methanol and acetonitrile (HPLC grade) were purchased from Merck KGaA, Darmstadt, Germany, while formic acid and ammonium formate (LC–MS grade) were obtained from Honeywell Trading Co., Ltd., Shanghai, China. PSA, C18, GCB, NH2, Al-A, Al-N, and Al-B were purchased from Beijing Naou Technology Co., Ltd., Beijing, China. GCB was purchased from Shanghai Anpu Experimental Technology Co., Ltd., Shanghai, China. 

Reference standards including clonidine, tadalafil, phenolphthalein, glibenclamide, nifedipine, pseudovardenafil, gliclazide, norneosildenafil, sildenafil, acetildenafil, dimethylreddenafil, and acetylvardenafil were purchased from Guangzhou Jiatu Technology Co., Ltd., Guangzhou, China. Bupropion, phenformin, hydroxyhomosildenafil, atenolol, alprazolam, triazolam, estazolam, and amobarbital were obtained from TMRM Quality Inspection Technology Co., Ltd., Tianjin, China. Tolazamide, glipizide, and noracetildenafil were purchased from Beijing Manhage Biotechnology Co., Ltd., Beijing, China. Prazosin, reserpine, rimonabant, and homosildenafil were purchased from Shanghai ANPEL-TRACE Standard Technical Service Co., Ltd., Shanghai, China. Diazepam, nitrazepam, lorazepam, zolpidem, and secobarbital were purchased from Tianjin Scientific Standard Co., Ltd., Tianjin, China. Orlistat, benfluorex, aininidipine, naturetin, alapuli, and candesartan cilexetil were obtained from Tianjin Alta Scientific Co., Ltd., Tianjin, China.

All reference standards had purities greater than 95%. Individual stock solutions (100 mg/L) were prepared in methanol and stored at −18 °C in the dark. Appropriate volumes of stock solutions were mixed and diluted with methanol to obtain a mixed standard solution at 1 mg/L. Working solutions were prepared by further dilution with blank matrix extracts and stored at 4 °C prior to analysis. All samples analyzed in this study were collected and provided by the Technical Center of Xiamen Customs. A total of 110 samples were included; each claimed to possess one or more functional properties, such as enhancing immunity, improving metabolism, promoting weight loss, supporting intestinal health, improving sleep quality, alleviating fatigue, regulating bodily functions, clearing heat and dampness, exerting anti-inflammatory and detoxifying effects, protecting the cardiovascular system, relieving cough and asthma, and assisting in the regulation of blood pressure and blood lipids. The sample types comprised solid beverages *(n* = 56), liquid beverages (*n* = 20), jellies (*n* = 17), tablets (*n* = 16), and oral liquid (*n* = 1).

### 2.3. Liquid Chromatography Conditions

Chromatographic separation was performed using a Kinetex F5 column. In positive-ion mode, the mobile phase consisted of 5 mmol/L ammonium formate with 0.1% formic acid in water (Mobile phase A) and methanol: acetonitrile (1:1, *v*/*v*) (Mobile phase B). In negative-ion mode, the aqueous phase (Mobile phase A) was ultrapure water, and the organic phase (Mobile phase B) was methanol: acetonitrile (1:1, *v*/*v*). The flow rate was set at 0.5 mL/min, the injection volume was 5 μL, and the column temperature was maintained at 40 °C. The gradient elution procedure is shown in Table 1. The same gradient profile was applied in both positive- and negative-ion modes, with differences only in mobile phase composition.

### 2.4. Mass Spectrometry Conditions

The X500R QTOF mass spectrometer was operated in both positive- and negative-ESI modes with IDA(). The ion source temperature was set to 550 °C, with a nebulizer gas pressure of 55 psi, an auxiliary heating gas pressure of 55 psi, an air curtain gas pressure of 35 psi, and voltages of 5500 V and −4500 V, respectively. The full scanning range for the first-stage TOF-MS is 50–1200 Da, while the accurate mass fragment scanning range for the second-stage TOF-MS/MS is also 50–1200 Da. The accumulation time was 0.10 s for TOF-MS and 0.05 s for TOF-MS/MS, resulting in a total cycle time of 0.662 s. The IDA trigger threshold was set at 100 cps. Dynamic background subtraction (DBS) was enabled. Collision energy is maintained at 40 ± 20 V.

### 2.5. Sample Pretreatment

Solid samples were ground and homogenized prior to analysis. Approximately 1.0 g of homogenized solid sample (accurate to 0.01 g) or 1.0 mL of liquid sample was transferred into a 50 mL PTFE centrifuge tube. Methanol (20 mL) was added, and the mixture was sonicated for 10 min. After cooling to room temperature, the extract was centrifuged at 8000 r/min for 5 min. The supernatant was transferred to a 25 mL volumetric flask and diluted to volume with methanol.

An aliquot of 1.5 mL of the extract was transferred into a 5 mL centrifuge tube, followed by the addition of 25 mg primary secondary amine (PSA) and 50 mg C_18_ sorbent. The mixture was vortexed for 1 min and centrifuged at 15,000 r/min for 5 min. The supernatant (1.0 mL) was filtered through a 0.22 μm nylon membrane prior to UPLC-Q-TOF-MS analysis.

### 2.6. Database Establishment and Screening Criteria

A single-compound standard solution at a concentration of 500 ng/mL was analyzed by UPLC-QTOF-MS. Data were acquired in IDA mode at collision energies of 20, 40, and 60 eV. Background ions were automatically removed using the DBS function.

A screening database was established containing compound names (Chinese and English), CAS numbers, precursor ion exact masses, and MS/MS spectra.

The positive identification criteria for 38 quality control compounds are as follows: On the basis of ensuring a signal-to-noise (S/N) ratio greater than 3, the standard for positive-confirmation parameters is: retention time error ≤ ±2.5%, mass error ≤ 5 × 10^−6^ (5 ppm), isotope pattern deviation ≤ 10%, and library score ≥ 80. These parameters were weighted at 30%, 30%, 10% and 30%, respectively, in the final identification score.

It is important to note that full quantitative validation (linearity, recoveries, precisions, SDLs, LOQs) was performed for the 38 quality control compounds, as described in Section 3.4. For the remaining 352 compounds in the screening database, which were not supported by reference standards during the validation phase, mass error ≤ 5 ppm, isotope deviation ≤ 10%, and library score ≥ 80 serve as the criteria for suspected screening. These parameters were weighted at 40%, 20% and 40%, respectively, in the final identification score.

### 2.7. Identification of Unknown Structural Analogues

Samples were prepared according to the procedure described in Section 2.5 and analyzed using IDA. Analyze the MS/MS spectra of known analogues, extract characteristic fragment ions of known scaffold structures to generate extraction ion chromatograms (XICs) to determine the retention time of unknown substances and use quasi-molecular ions to determine the molecular formula. Obtain MS/MS spectra of precursor ions to elucidate characteristic neutral losses and fragment ions, in order to infer the core scaffold structure and substituents. Characteristic fragment ions of PDE-5 inhibitor analogues are summarized in Table 2.

### 2.8. Data Processing

All data acquisition and processing were performed using SCIEX OS Software (Version 1.7.0.36606). Data visualization and plotting were carried out using Origin 2024 and ChemDraw 2.1.1.

## 3. Results

### 3.1. Database Establishment and Basis for the Selection of Representative Quality Control Compounds

A comprehensive in-house screening database comprising 390 non-edible substances was constructed to support both suspected and non-targeted screening workflows. The compounds were selected from multiple sources to ensure broad applicability and regulatory relevance. The compounds were primarily derived from official regulatory lists issued by the State Administration for Market Regulation of China, specifically the supplementary testing methods (BJS series) and the national food safety standards for the determination of non-edible substances in food, as well as from the peer-reviewed literature reporting cases of food adulteration and emerging non-edible substances in functional foods, with an emphasis on compounds frequently detected in solid beverages, tablets, and other high-risk product categories. The database encompasses both internationally reported non-edible substances (e.g., sildenafil, tadalafil, phenolphthalein) and compounds specifically relevant to the Chinese regulatory context, thereby aligning with domestic surveillance priorities and global food safety concerns.

To comprehensively validate the analytical method, 38 quality control compounds were strategically selected from the 390-compound database to represent the full range of physicochemical properties encountered in the broader compound list. On the one hand, as representatives of the major pharmacological categories present in the database, the selected compounds encompass antihypertensives, hypoglycemics, weight-loss agents, sedatives–hypnotics, and antifatigue compounds, ensuring coverage of the primary regulatory concerns. On the other hand, to ensure the method is applicable across a wide range of chemical structures, chemical diversity was taken into consideration, ensuring coverage of *m/z* from 206 to 609, a logP range from –0.16 to 7.12, and retention times uniformly distributed from 3.80 min to 20.86 min, with the inclusion of two pairs of isomers (pseudovardenafil and norneosildenafil; acetildenafil, dimethylacetildenafil, and acetylvardenafil) to assess chromatographic resolution. Additionally, the ionization mode distribution (32 ESI+ and 6 ESI−) was selected to mirror that of the full database (Table S1 and Table 3). Through this systematic selection, the 38 quality control compounds comprehensively represent the physicochemical diversity of the full 390-compound database. The successful validation of these representative compounds provides high confidence that the method exhibits comparable analytical performance across the entire database.

### 3.2. Optimization of Chromatographic Conditions

The chromatographic conditions were systematically optimized using three difficult-to-separate isomeric compounds—acetildenafil, dimethyl acetildenafil, and acetylvardenafil—selected from the quality control set as model analytes. With methanol as the organic phase, the effect of ammonium formate concentration in the aqueous phase was investigated. Varying the concentration from 2.5 to 10 mmol/L markedly influenced chromatographic behavior, among which 5 mmol/L ammonium formate provided the most favorable compromise between retention and separation. Accordingly, 5 mmol/L ammonium formate was selected for subsequent optimization. Methanol and acetonitrile were then evaluated as organic phases. Although acetonitrile afforded improved peak shape and signal response, satisfactory separation of the target isomers was not achieved. To balance peak shape, response, and separation, a methanol–acetonitrile mixture was further assessed. At a 1:1 (*v*/*v*) ratio, three chromatographic peaks were clearly detected, although baseline separation was not fully achieved. Given the significant influence of mobile-phase pH on chromatographic separation, formic acid was added to the aqueous phase at concentrations ranging from 0.01% to 0.1%. The addition of 0.1% formic acid resulted in the optimal separation performance. Consequently, the final chromatographic conditions were established as follows: in positive-ion mode, the mobile phase consisted of 5 mmol/L ammonium formate containing 0.1% formic acid in water and methanol: acetonitrile (1:1, *v*/*v*); in negative-ion mode, water and methanol: acetonitrile (1:1, *v*/*v*) were employed. Representative chromatograms obtained at each optimization stage are shown in Figure S1A–H.

### 3.3. Optimization of Mass Spectrometry Conditions

Following the established optimal chromatographic parameters, a mixed standard solution was prepared as detailed in Section 2.2 and subsequently analyzed in both ESI+ and ESI− modes. The results showed that thirty-two compounds exhibited predominant signal intensity in ESI+ mode, whereas the remaining six compounds demonstrated superior intensity in ESI− mode. The chromatograms of 38 quality control compounds are shown in Figure S2. High-resolution mass spectrometric data for all 38 quality control compounds are detailed in Table 3, HRMS data information of quality control compounds.

### 3.4. Pretreatment Optimization Analysis

#### 3.4.1. Optimization of Solvent Extraction

Blank samples were spiked with standard solutions to obtain a final concentration of each target compound of 30 ng/mL. In the analysis of non-edible substances in food matrices, methanol and acetonitrile—used either as pure solvents or in mixtures with water or acid solutions—are commonly employed as extraction solvents [27,28,29]. In this study, methanol and acetonitrile were evaluated as extraction solvents. Using an average recovery range of 60–120% as the acceptability criterion, twelve representative compounds were selected to assess the extraction performance of each solvent (Figure S3). The results demonstrated that methanol consistently provided higher extraction recoveries for the target compounds compared with acetonitrile. This superior performance is likely attributed to the higher polarity of methanol, which facilitates more efficient extraction through mechanisms such as hydrogen bonding and van der Waals interactions [30]. In addition to its superior extraction efficiency, methanol also offers advantages over acetonitrile in terms of safety and cost. Therefore, methanol was selected as the extraction solvent for all subsequent experiments in this study.

#### 3.4.2. Optimization of Extraction Method

Optimization of sample pretreatment is essential to ensure complete dissolution of target compounds and to maximize extraction efficiency for subsequent analysis. In this study, methanol was used as the extraction solvent to systematically evaluate and compare two commonly employed techniques—vortex mixing and ultrasound-assisted extraction—with respect to their extraction efficiency for the target compounds. Using the average recovery rate of 60–120% as the acceptability criterion, twelve representative compounds were selected to assess the performance of each extraction method (Figure S4). Ultrasound-assisted extraction operates via cavitation, where the formation and violent collapse of microscopic bubbles generate localized high pressure and temperature. This process significantly enhances solvent penetration into the sample matrix, improves interfacial mass transfer, and promotes analyte desorption and dissolution [31]. In contrast, vortex mixing primarily relies on mechanical shear and convective forces, which generally provide lower extraction intensity and more limited penetration depth than ultrasonication [32]. The results indicated that although both methods effectively extracted the target compounds, recoveries obtained by vortex mixing were consistently lower than those achieved with ultrasound-assisted extraction. Notably, the ultrasound-assisted method demonstrated superior overall performance, yielding higher average recoveries with improved stability and reproducibility across replicate samples.

Based on these findings, ultrasound-assisted extraction was selected as the standardized pretreatment protocol. This choice enhances overall method sensitivity, reproducibility, and analytical throughput, thereby establishing a robust foundation for the detection of nonfood substances in complex food matrices.

#### 3.4.3. Optimization of Extraction Time

Following the selection of methanol as the extraction solvent and ultrasonication as the extraction mode, the extraction time was systematically optimized as a key parameter influencing the efficiency of target compound recoveries. Insufficient sonication time prevents the extraction system from reaching equilibrium, resulting in incomplete recovery as a portion of the analytes may remain in the pellet after centrifugation. Conversely, excessive sonication unnecessarily prolongs the pretreatment cycle and increases co-extraction of matrix interferents through enhanced solubilization of impurities, thereby elevating matrix effects and complicating subsequent cleanup [33,34]. Therefore, the average recoveries of target compounds were evaluated at sonication durations of 10, 20, 30, 40, and 50 min. Using the average recovery rate of 60–120% (n = 3) as the acceptability criterion, twelve representative compounds were selected to evaluate the performance of each extraction time (Figure S5). The results indicated that satisfactory recoveries for all target compounds were achieved within 10 min of ultrasonication. Further extension of sonication time did not result in a significant overall improvement in average recovery. Balancing analytical efficiency and effectiveness, a sonication time of 10 min was selected as optimal.

#### 3.4.4. Selection of Purification Agent Types

This study evaluated the purification efficacy of seven DSPE adsorbents, namely Al-A, Al-N, Al-B, C_18_, NH_2_, GCB, and PSA. Among these, PSA, NH_2_, Al-A, Al-B, and Al-N possess amino functional groups and are primarily employed to remove polar interferences, including organic acids, sugars, phenols, and certain pigments. C_18_, a reversed-phase adsorbent, effectively retains nonpolar and moderately polar compounds, such as lipids, sterols, waxes, and nonpolar pigments. GCB exhibits a strong affinity for planar molecules, particularly pigments, due to its highly ordered graphitic surface structure [35,36]. Using an average recovery rate of 60–120% as the acceptability criterion, twelve representative compounds were selected to evaluate the performance of each purification strategy (Figure S6). The results demonstrated that C_18_ provided the optimal overall recoveries among the seven tested adsorbents, followed by PSA, NH_2_, Al-A, Al-B, and Al-N. GCB yielded the poorest recoveries, particularly for hypoglycemic agents such as glibenclamide and antifatigue agents such as sildenafil. The strong adsorption of these compounds by GCB is attributed to efficient π–π stacking interactions between their electron-rich planar rings and the graphitic surface of the adsorbent [37]. Although sulfonylurea drugs possess less overall planarity, the presence of aromatic rings in their structures still facilitates detectable, albeit weaker, interactions with GCB. Recoveries exceeding 120% were observed for certain compounds when using the amino-functional groups (NH_2_, Al-A, Al-B, Al-N), potentially due to their limited capacity to remove lipids and sugars, leading to significant matrix enhancement effects. Conversely, PSA showed pronounced adsorption of some sulfonylurea drugs, resulting in recoveries below 60%. This may be attributed to the use of an excessive PSA amount, where the adsorbent, after removing matrix interferences, retained additional capacity to strongly retain the target analytes, hindering their elution. Given the complexity and variability of food matrices, which contain pigments, fats, and sugars that can interfere with detection, contaminate instrumentation, and reduce column lifetime, effective cleanup is essential. Furthermore, combined adsorbents often provide superior purification compared to single adsorbents. Therefore, based on their complementary purification profiles and favorable recovery performance, C_18_ and PSA were selected as the optimal combination for further optimization of adsorbent dosage in subsequent experiments. Although the sample extract used for DSPE cleanup was composed entirely of methanol (1.5 mL of extract diluted to 25 mL with methanol), C_18_ still effectively retained nonpolar interferents such as lipids, sterols, and nonpolar pigments. This is attributed to the limited solubility of these compounds in pure methanol, which drives their partitioning onto the hydrophobic surface of the C_18_ sorbent. The effectiveness of C_18_ under these conditions was supported by the visibly cleaner extracts after treatment and by the low matrix effects observed for most target compounds (Table 4). PSA, containing primary and secondary amine groups, acted as a weak anion exchange sorbent. In the pure methanol matrix, the amine groups remained partially protonated and retained acidic interferents (e.g., free fatty acids, organic acids, polyphenols) via hydrogen bonding and dipole–dipole interactions. The complementary roles of C_18_ and PSA—removing nonpolar and polar interferents, respectively—ensured effective cleanup across the diverse food matrices analyzed in this study.

#### 3.4.5. PSA and C_18_ Purification Agent Dosage

This study systematically evaluated the effect of varying PSA and C_18_ sorbent dosages (25, 50, 75, 100, and 125 mg) on the recovery of target compounds. Using an average recovery rate ranging from 60% to 120% as the acceptability criterion, twelve representative compounds were selected to assess the performance of each purification agent dosage (Figures S7 and S8). The findings revealed that at a PSA dosage of 25 mg, recoveries for the majority of target compounds exceeded 70%. However, upon increasing the PSA dosage, compound-dependent trends in recovery were observed. Notably, the recovery rates of sulfonylurea compounds significantly decreased, aligning with prior speculation regarding their low recovery (below 60%) when exposed to higher PSA amounts. Conversely, with increasing C_18_ dosages, recoveries typically demonstrated an initial enhancement followed by a subsequent reduction. Specifically, a C_18_ dosage of 50 mg ensured that recoveries for all target compounds met the acceptability criterion, with no significant performance improvement observed at higher sorbent amounts. Following the principles of green economy, which favor minimal sorbent usage without compromising cleanup efficacy, the optimal combination of purification agents was determined to be 25 mg of PSA and 50 mg of C_18_.

#### 3.4.6. Matrix Effect Evaluation

The matrix effect (ME) is a prevalent interference phenomenon encountered in the detection of trace substances during mass spectrometry analysis. The intensity of matrix effects can be systematically classified according to the absolute value of |ME|: |ME| ≤ 20% represents a weak matrix effect, 20% < |ME| ≤ 50% indicates a moderate matrix effect, and |ME| > 50% is classified as a strong matrix effect [37]. This study examined four distinct food matrices—tablet, liquid beverage, solid powder, and oral liquid—for injection analysis. The matrix effect was quantitatively assessed using the formula: matrix effect (ME%) = [((slope of matrix-matching standard curve)/(slope of pure solvent standard curve)) − 1] × 100%. The specific results are presented in Table 4. In mass spectrometry analysis, commonly employed methods for compensating for matrix effects include the isotope internal standard method and the matrix-matching standard solution method. Given the current challenges in obtaining commercial isotope internal standard solutions that encompass all target substances, this study utilized matrix matching.

### 3.5. Methodological Evaluation

#### 3.5.1. Linear Range, Standard Curve, R^2^, SDLs and LOQs

Under the optimal instrument analysis conditions, a series of standard working solutions spanning concentrations from 0.005 to 5.0 mg/kg (0.005, 0.05, 0.125, 0.25, 0.5, 1.25, 2.5, 5.0 mg/kg) were prepared to assess the linearity of the method. Matrix-matched calibration standards were prepared by spiking appropriate volumes of the mixed standard solution into a blank matrix, followed by stepwise dilution. Calibration curves were then constructed by plotting analyte concentration (*x*-axis) against the corresponding peak area (*y*-axis). The results demonstrated exceptional linearity for all target compounds within the specified range, with R^2^ > 0.99. The SDLs and LOQs of the method were determined by incorporating mixed standard solutions with varying concentrations into matrix samples. Detailed results are summarized in Table 5. In accordance with SANTE/11312/2021 guidelines, the SDL is defined as the lowest mass concentration detectable at various spiking levels. Conversely, the lowest spiked level with S/N ≥ 10 is considered the LOQ of the compound [38]. Results indicate that 92.1% of the quality control compounds exhibited SDLs within the range of 0.003–0.1 mg/kg, whereas 7.9% were between 0.1 and 0.5 mg/kg, demonstrating the method’s high sensitivity. Considering that the concentration of illegal additives in actual samples is typically elevated, this method is particularly well-suited for the non-targeted screening of non-edible substances in food. It is important to note that the method for determining the screening limit in this study diverges from the traditional approach based on S/N ≥ 3. Although a direct mathematical correlation between SDLs and LOQs is not explicitly established by this method, all determined SDLs consistently remain below the corresponding matrix-matched LOQs [39].

#### 3.5.2. Method Precision and Spiked Recovery Rates

This study examined the recovery rates of the target substance across diverse sample matrices at spiked concentrations of 0.1, 0.2, and 1.0 mg/kg. The external standard method was employed to quantitatively assess each concentration, facilitating the calculation of the average recovery rates (*n* = 6) and RSD to evaluate the method’s accuracy and precision. As presented in Table 6, the average recovery rates for tablets ranged from 70.09% to 110.93%, accompanied by RSD values spanning 0.71% to 7.66%. For liquid beverages, the average recovery rates were observed between 69.76% and 102.5%, with corresponding RSD values from 0.67% to 6.30%. Solid beverages exhibited average recovery rates ranging from 77.58% to 103.67%, with RSD values from 0.23% to 8.20%. Oral liquids yielded average recovery rates ranging from 67.79% to 113.59%, with RSD values between 0.74% and 9.37%. Overall, the average recovery rates for spiked samples across different matrices fell within the acceptable range of 60% to 120%, with RSD values ≤ 20%. Consequently, this method exhibits satisfactory precision and is well-suited for the rapid screening of non-edible substances in food matrices.

## 4. Development of a Screening and Identification Workflow

**Figure 1:**
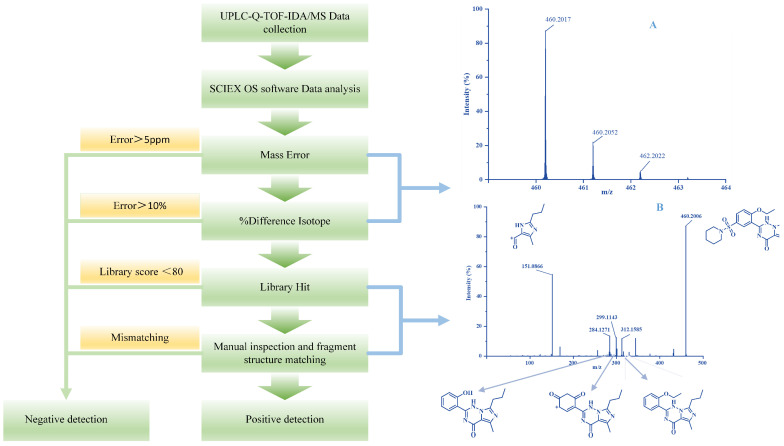
Suspected screening strategy process.

**Figure 2:**
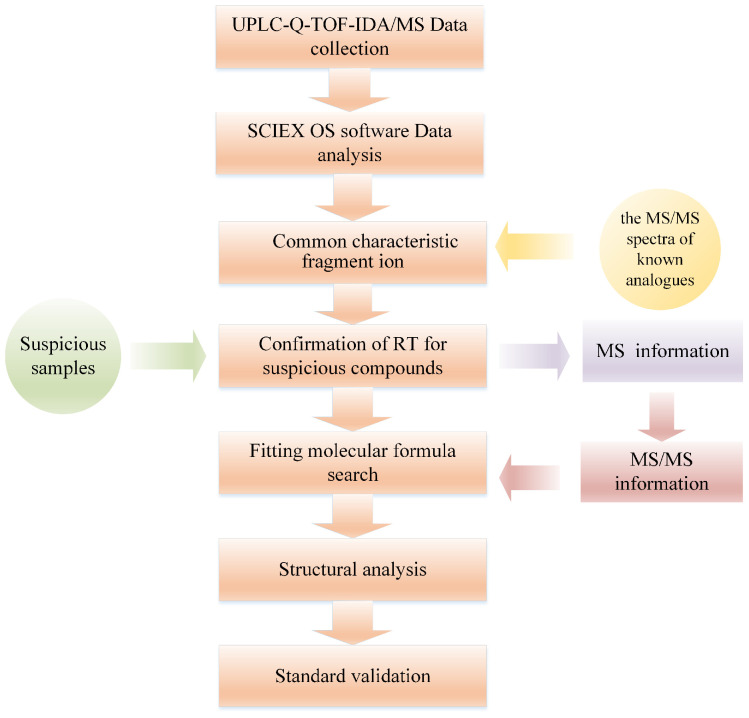
Non-targeted identification strategy process.

The research findings consistently demonstrate that this screening method possesses both stability and high sensitivity, making it suitable for the non-targeted screening of non-edible substances in food matrices. Subsequent to sample pretreatment, the method is applicable for UPLC-Q-TOF-MS analysis. As an illustrative example, the screening of pseudovardenafil was utilized to demonstrate the non-targeted screening workflow for the analysis of non-edible substances. The data was imported into SCIEX OS software for analysis, the molecular formula was input into the screening list, and the ionization mode was defined as [M+H]^+^ to obtain the XIC, MS, and MS/MS spectrum of the suspicious substance. Firstly, the information was matched in the MS spectrum, with a precursor exact-mass error of less than 5 ppm and an isotope distribution deviation of less than 10%, as shown in Figure 1A. Due to the successful matching of the first-level precise-mass number and isotope distribution of the suspicious target peak with the pseudo vardenafil, further matching the MS/MS spectra of pseudo vardenafil in the database resulted in a matching score of 92.2 points. Finally, based on the structural formula of pseudo vardenafil, the five main fragment structures were completely matched, as shown in Figure 1B. It was determined to be a positive detection. The comprehensive screening workflow, applied to the actual sample database within this study, is delineated in Figure 1, while the strategy for unknown analyte identification is presented in Figure 2.

### Sample Screening

This study involved the selection of 110 batches of commercially available samples collected from the Xiamen Customs Technical Center. The samples were analyzed using the method established by our research institute. Positive samples were screened and quantitatively assessed through the external standard method.

Phenolphthalein (7.82 mg/g) was identified in a solid beverage. Lacidipine (68.15 mg/g) was identified in a solid beverage. Sennoside A (143.08 mg/g) and sennoside B (69.39 mg/g) were detected in an enzyme beverage. A sample of enzyme jelly was found to contain deacetylbisacodyl (8.54 mg/g). Theophylline (14.51 mg/g) was detected in a protein nutrient solid beverage devoid of tea ingredients. Atropine (0.16 mg/g) and scopolamine (0.09 mg/g), both tropane alkaloids, were concurrently identified in an alcoholic beverage. A tablet was found to contain higenamine (63.65 mg/g), a member of the benzylisoquinoline alkaloid class. Acetaminophen (204.54 mg/g) was identified in a tablet sample. Currently, research has shown that it is often illegally added to plant-based beverages claiming to have anti-inflammatory and pain-relieving properties [40]. Aurantio-obtusin (18.15 mg/g), a lipophilic anthraquinone compound extracted from Semen Cassiae, was detected in solid drinks. Research has demonstrated that it can induce diarrhea, lower blood pressure, and reduce blood lipids. It is commonly employed in the treatment of obesity, diabetes, and related complications [41]. Tadalafil (227.12 mg/g) was identified in a tablet sample. The unauthorized inclusion of these substances in functional foods purporting to offer benefits like “antifatigue” and “enhancing sexual function” remains prevalent. Prolonged consumption could significantly jeopardize consumer health, making it a primary focus for regulatory efforts in food safety oversight [42].

**Figure 3:**
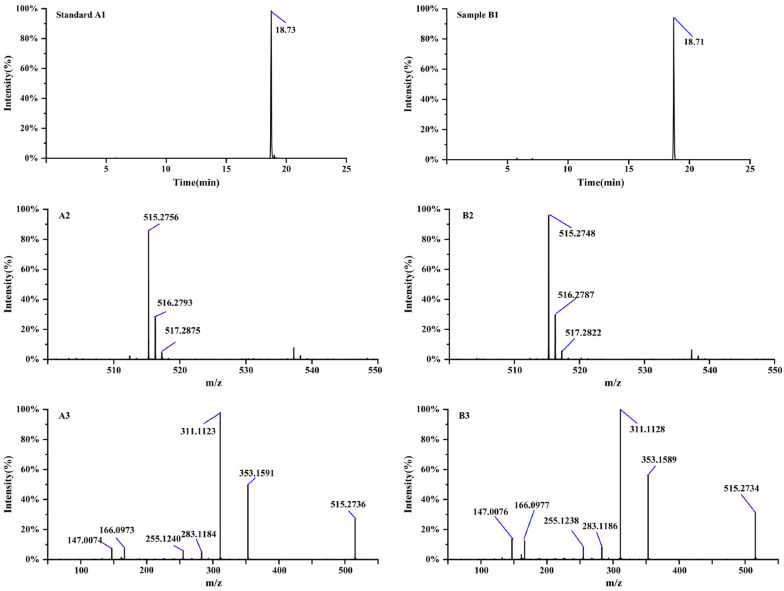
Comparison of suspicious substances in the sample with standard information. (**A1**) Extracted ion chromatogram of the reference standard; (**A2**) MS^1^ spectrum of the reference standard; (**A3**) MS^2^ spectrum of the reference standard; (**B1**) Extracted ion chromatogram of the unknown compound in the sample; (**B2**) MS^1^ spectrum of the unknown compound in the sample; (**B3**) MS^2^ spectrum of the unknown compound in the sample.

In addition, the unknown identification strategy developed in this study was applied to real-sample analysis, successfully identifying a structural analogue of carbodenafil in a tablet sample. The suspect compound was detected at a retention time of 18.71 min, as shown in Figure 3(B1). Primary full-scan MS data at this retention time revealed a quasi-molecular ion [M+H]^+^ at *m/z* 515.2748, as shown in Figure 3(B2). Molecular-formula fitting of this ion using MS analysis software (SCIEX OS Software (Version 1.7.0.36606)) suggested a composition of C_29_H_34_N_6_O_3_. Subsequent acquisition of its MS/MS spectrum information showed common fragment ions with carbodenafil analogues, including *m/z* 147.0076, 166.0977, 255.1238, 283.1186, and 311.1128, indicating that it shares the core structure with carbodenafil analogues, as shown in Figure 3(B3).

**Figure 4:**
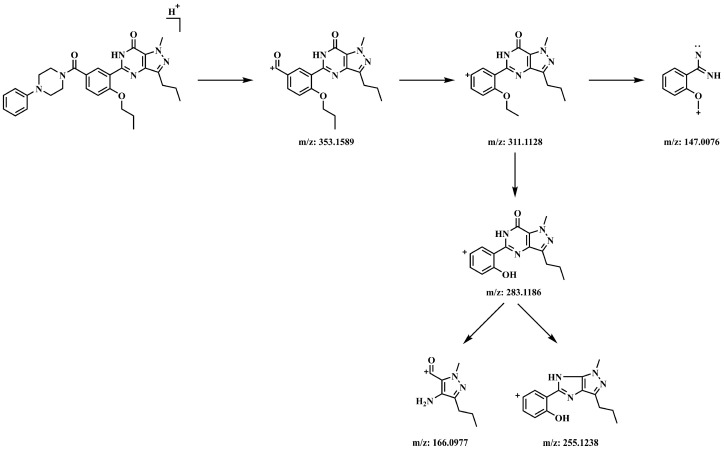
The fragmentation pattern of suspicious substances in the sample by mass spectrometry. Note: the “..” above the nitrogen atom in the figure represents a lone pair of electrons.

A ChemSpider search using the proposed molecular formula (C_29_H_34_N_6_O_3_) indicated that the suspect compound was likely phenyl 3-desethyl 3-propyl carbodenafil. This preliminary assignment was further supported by structural elucidation of its MS/MS spectrum. A key characteristic fragment ion was observed at *m/z* 353.1589, which is 14 Da higher than a common fragment ion (*m/z* 339) found in typical carbodenafil analogues. The mass difference corresponds precisely to an additional methylene (-CH_2_-) unit, consistent with the proposed phenylpropyl substitution on the core scaffold. The identity of the compound was conclusively confirmed by comparison with an authentic phenylpropyl carbodenafil standard, which showed identical chromatographic retention time and matching multi-level MS/MS fragmentation patterns. The comparison information between suspicious substances in the sample and standard samples XIC, MS, and MS/MS is shown in Figure 3. The fragmentation pattern of suspicious substances in the sample by mass spectrometry is shown in Figure 4.

In summary, among the 110 actual samples analyzed, a total of 13 non-edible substances were detected, corresponding to a detection rate of 11.81%. The positive samples involved multiple product categories, including solid beverages (four samples), jellies (one sample), liquid beverages (three samples), and tablets (four samples).

For most non-edible substances, a clear association was observed between the detected compounds and the claimed product functions. Compounds associated with weight-loss effects—such as phenolphthalein, sennoside A, sennoside B, and deacetylbisacodyl—were predominantly detected in solid beverages and enzyme jellies marketed with weight-loss claims. Similarly, tadalafil was identified in tablets labeled with antifatigue and sexual-enhancement functions. These findings suggest that the illegal addition of most non-edible substances aligns with the purported functional benefits of the products in which they were found. However, the study also identified notable exceptions that underscore the limitations of function-oriented targeted screening. Atropine and scopolamine—tropane alkaloids with potent sedative, hypnotic, and amnesic effects—were detected in an alcoholic beverage marketed with claims of “immunity enhancement” and “fatigue relief.” The pharmacological effects of these compounds are unrelated to the claimed functions of the product. Instead, unscrupulous manufacturers may exploit the initial excitement, disorientation, and subsequent sedation and amnesia induced by these substances to mimic the effects of ordinary alcohol intoxication, thereby concealing the illicit addition and their underlying intent. This case exemplifies how non-edible substances may be added for ulterior motives unrelated to product efficacy, further demonstrating that targeted screening methods relying solely on product label claims carry a substantial risk of omission.

The detection of both function-aligned and function-unrelated non-edible substances across multiple product categories highlights the critical need for broad-spectrum, non-targeted screening methodologies in routine food safety surveillance.

## 5. Conclusions

This study successfully developed a UPLC-Q-TOF-MS method, integrating DSPE pretreatment and an IDA mode, for the comprehensive high-throughput screening of 390 non-edible substances in food. Under the systematically optimized conditions, the DSPE pretreatment efficiently cleans up complex food matrices while simultaneously extracting multi-category non-edible substances with diverse physicochemical properties. Rigorous validation across four representative food matrices confirmed the method’s reliability and applicability, with all key analytical parameters—including ME, linearity, recoveries, precisions, SDLs and LOQs—fully complying with established criteria. Furthermore, by systematically elucidating the mass spectrometric fragmentation pathways of PDE-5 inhibitor analogues, a novel non-targeted identification strategy for unknown structural analogues was developed, substantially enhancing the method’s capability for detecting emerging non-edible substances. The developed method was subsequently applied to 110 commercially sourced food samples obtained from diverse regions. This application led to the identification of 12 known non-edible substances via database matching. Moreover, by employing the aforementioned unknown-compound identification strategy, a novel carbodenafil analogue—phenyl 3-desethyl 3-propyl carbodenafil—was successfully identified. These findings reveal a potential risk of illegal addition in some commercially available conventional foods marketed with specific functional claims (often described as containing only natural ingredients), suggesting that regulatory authorities should strengthen monitoring and control of such products. The detection of atropine and scopolamine in an alcoholic beverage marketed with unrelated functional claims highlights the limitations of function-oriented targeted screening and underscores the necessity of broad-spectrum, non-targeted approaches. By integrating database-driven suspected screening with fragmentation pattern-based non-targeted identification, this work provides a practical analytical platform that effectively bridges the regulatory gap posed by emerging structural analogues, offering an efficient solution for high-throughput screening of non-edible substances and serving as a robust tool for advancing food safety monitoring and risk assessment.

## Figures and Tables

**Table 1:** Gradient elution program.

Time (min)	Flow (mL/min)	Mobile Phase A (%)	Mobile Phase B (%)
0.0	0.5	95	5
1.0	0.5	95	5
8.0	0.5	55	45
17.0	0.5	40	60
20.0	0.5	5	95
22.0	0.5	5	95
22.1	0.5	95	5
25.0	0.5	95	5

**Table 2:** Common characteristic fragment ion.

Category	Common Characteristic Fragment Ion
Sildenafil analogues	*m/z* 377; *m/z* 311; *m/z* 283; *m/z* 255
Vardenafil analogues	*m/z* 376; *m/z* 377; *m/z* 312; *m/z* 299; *m/z* 284; *m/z* 169; *m/z* 151
Hongdenafil analogues	*m/z* 353; *m/z* 341; *m/z* 325; *m/z* 311; *m/z* 297; *m/z* 166
Carbodenafil analogues	*m/z* 339; *m/z* 311; *m/z* 283; *m/z* 255; *m/z* 166; *m/z* 147
Tadalafil analogues	*m/z* 302; *m/z* 274; *m/z* 262; *m/z* 250; *m/z* 204; *m/z* 197; *m/z* 169; *m/z* 135

**Table 3:** HRMS data information of quality control compounds.

Index	Compounds	Formula	Retention Time	Adduct/Charge	Precursor Mass	Found at Mass	Mass Error	Fragment Mass			
1	Clonidine	C_9_H_9_Cl_2_N_3_	4.45	[M+H]^+^	230.025	230.0245	−0.5	212.9986	194.0482	159.9719	132.9609
2	Tadalafil	C_22_H_19_N_3_O_4_	10.60	[M+H]^+^	390.145	390.1447	−0.4	268.1079	262.0861	169.0758	135.0439
3	Phenolphthalein	C20H14O4	9.77	[M+H]^+^	319.096	319.0963	−0.6	225.0544	197.0598	141.0702	105.0333
4	Glibenclamide	C_23_H_28_ClN_3_O_5_S	16.21	[M+H]^+^	494.151	494.1511	0.0	395.0474	369.0671	304.0735	169.0051
5	Nifedipine	C_17_H_18_N_2_O_6_	11.96	[M+H]^+^	347.124	347.1238	0.2	254.1042	241.0972	195.0913	168.0804
6	Pseudovardenafil	C_22_H_29_N_5_O_4_S	15.41	[M+H]^+^	460.201	460.2017	0.8	432.1708	344.1483	284.1271	151.0866
7	Gliclazide	C_15_H_21_N_3_O_3_S	11.86	[M+H]^+^	324.138	324.1375	−0.5	168.1131	153.1024	127.1229	110.0961
8	Norneosildenafil	C_22_H_29_N_5_O_4_S	17.29	[M+H]^+^	460.201	460.2011	−0.5	469.2006	311.1506	283.1191	256.0957
9	Sildenafil	C_22_H_30_N_6_O_4_S	9.97	[M+H]^+^	475.212	475.2119	−0.7	311.1503	299.1141	283.1190	99.0916
10	Acetildenafil	C_25_H_34_N_6_O_3_	8.45	[M+H]^+^	467.277	467.2762	−0.7	396.2026	353.1608	297.1341	127.1229
11	Dimethyl acetildenafil	C_25_H_34_N_6_O_3_	9.38	[M+H]^+^	467.277	467.2762	−0.7	410.2193	353.1612	297.1351	127.1235
12	Acetylvardenafil	C_25_H_34_N_6_O_3_	9.05	[M+H]^+^	467.277	467.2762	−0.6	325.1308	341.1619	297.1352	127.1231
13	Bupropion	C_13_H_18_ClNO	8.03	[M+H]^+^	240.115	240.1147	−1.2	166.0417	139.0310	130.0649	103.0542
14	Phenformin	C_10_H_15_N_5_	4.98	[M+H]^+^	206.14	206.1399	−0.6	206.1402	147.0921	130.0653	105.0698
15	Hydroxyhomosildenafil	C_23_H_32_N_6_O_5_S	9.86	[M+H]^+^	505.223	505.228	0.1	487.2126	377.1275	311.1514	283.1200
16	Atenolol	C_14_H_22_N_2_O_3_	3.80	[M+H]^+^	267.17	267.1701	−0.8	225.1239	190.0866	145.0650	133.0651
17	Prazosin	C_19_H_21_N_5_O_4_	7.71	[M+H]^+^	384.167	384.1661	−1.4	368.1352	247.1184	231.0873	138.0550
18	Reserpine	C_33_H_40_N_2_O_9_	14.39	[M+H]^+^	609.281	609.2801	−1.0	448.1976	397.2126	265.1867	195.0655
19	Rimonabant	C_22_H_21_C_l3_N_4_O	19.29	[M+H]^+^	463.085	463.085	−0.7	380.9956	362.9841	300.0220	164.0261
20	Tolazamide	C_14_H_21_N_3_O_3_S	10.69	[M+H]^+^	312.138	312.1374	−0.8	172.0421	155.0161	141.1021	115.1227
21	Glipizide	C_21_H_27_N_5_O_4_S	10.97	[M+H]^+^	446.186	446.1857	−0.6	347.0813	321.1015	286.0644	167.0161
22	Orlistat	C_29_H_53_NO_5_	20.86	[M+H]^+^	496.4	496.3986	−2.1	337.3095	319.2991	160.0967	114.0911
23	Benfluorex	C_19_H_20_F_3_NO_2_	13.3	[M+H]^+^	352.152	352.1518	−0.2	230.1152	187.0733	159.0417	149.0599
24	Diazepam	C_16_H_13_ClN_2_O	11.65	[M+H]^+^	285.079	285.0789	0.0	257.0853	222.1155	193.0987	154.0419
25	Noracetildenafil	C_24_H_32_N_6_O_3_	8.83	[M+H]^+^	453.261	483.2609	0.1	396.2037	297.1349	285.1351	113.1075
26	Alprazolam	C_17_H_13_ClN_4_	10.2	[M+H]^+^	309.09	309.0901	−0.3	281.0761	274.1216	205.0762	165.0215
27	Triazolam	C_17_H_12_Cl_2_N_4_	10.21	[M+H]^+^	343.051	343.0512	−0.1	315.0327	253.0651	239.0374	165.0215
28	Nitrazepam	C_15_H_11_N_3_O_3_	9.72	[M+H]^+^	282.087	282.0871	−0.6	268.0848	236.0947	207.0920	180.0811
29	Lorazepam	C_15_H_10_Cl_2_N_2_O_2_	9.58	[M+H]^+^	321.019	321.0193	0.2	303.0087	275.0135	229.0526	163.0054
30	Zolpidem	C_19_H_21_N_3_O	7.77	[M+H]^+^	308.176	308.1750	−2.3	263.1169	235.1223	221.1069	145.0760
31	Homosildenafil	C_23_H_32_N_6_O_4_S	10.23	[M+H]^+^	489.228	489.2279	0.2	461.1977	283.1196	311.1506	99.0916
32	Estazolam	C_16_H_11_ClN_4_	9.57	[M+H]^+^	295.075	295.0743	−0.8	267.0554	241.0530	205.0757	165.0218
33	Secobarbital	C_12_H_18_N_2_O_3_	9.17	[M−H]^−^	237.124	237.1244	−0.3	194.1177	150.1277	108.082	85.0044
34	Amobarbital	C_11_H_18_N_2_O_3_	8.68	[M−H]^−^	225.124	225.1243	−0.7	182.1179	138.1280	96.9809	85.0047
35	Cilnidipine	C_27_H_28_N_2_O_7_	19.28	[M−H]^−^	491.182	491.1813	−2.2	357.1075	237.0659	208.0968	122.0242
36	Naturetin	C_15_H_14_F_3_N_3_O_4_S_2_	10.61	[M−H]^−^	420.031	420.0288	−4.2	327.9667	223.9929	196.982	160.0373
37	Alapuli	C_20_H_26_N_2_O_5_S	6.32	[M−H]^−^	405.149	405.1478	−3.0	363.1379	329.1506	217.134	164.0718
38	CandesartanCilexetil	C_33_H_34_N_6_O_6_	17.87	[M−H]^−^	567.152	567.1500	−3.8	411.1147	383.1136	340.1081	113.0404

**Table 4:** ME (%) evaluation of 38 quality control compounds in diverse food matrices.

Index	Compounds	ME (%)			
		Tablets	Liquid Beverages	Oral Liquids	Solid Beverages
1	Clonidine	7.17	4.69	−29.13	−24.71
2	Tadalafil	0.39	0.36	6.9	−0.29
3	Phenolphthalein	1.13	8.94	11.89	−5.96
4	Glibenclamide	3.28	12.79	3.7	−7.50
5	Nifedipine	2.52	7.97	−2.7	−6.21
6	Pseudovardenafil	1.29	14.37	16.7	−1.27
7	Gliclazide	1.89	9.61	11.26	6.17
8	Norneosildenafil	0.78	13.37	0.43	−2.15
9	Sildenafil	2.77	12.11	3.50	−3.59
10	Acetildenafil	1.49	8.26	−21.38	8.96
11	Dimethyl acetildenafi	2.67	14.44	−12.20	0.80
12	Acetylvardenafil	3.03	26.47	−0.63	6.45
13	Bupropion	8.10	8.86	−20.64	−20.29
14	Phenformin	7.60	6.67	−39.07	−25.97
15	Hydroxyhomosildenafil	0.19	12.06	10.22	−0.95
16	Atenolol	6.27	5.86	−31.76	−25.16
17	Prazosin	1.68	9.56	−17.25	−17.22
18	Reserpine	11.37	1.85	11.57	0.19
19	Rimonabant	2.18	17.09	21.66	19.55
20	Tolazamide	2.07	17.21	21.66	21.34
21	Glipizide	1.75	17.98	9.91	−0.20
22	Orlistat	28.63	10.65	−6.36	−1.47
23	Benfluorex	6.01	18.95	32.62	4.26
24	Diazepam	12.46	1.84	−21.23	−21.07
25	Noracetildenafil	1.32	13.25	−8.19	−7.05
26	Alprazolam	1.83	5.75	−8.49	−12.65
27	Triazolam	5.05	6.55	−5.27	−8.87
28	Nitrazepam	2.46	6.36	−11.84	−14.15
29	Lorazepam	1.50	12.29	10.38	−3.16
30	Zolpidem	5.26	11.03	−25.43	−18.66
31	Homosildenafil	0.75	15.24	9.73	−5.65
32	Estazolam	1.73	6.88	−9.71	−12.07
33	Secobarbital	2.37	6.42	4.55	−13.92
34	Amobarbital	2.42	7.04	5.95	−12.17
35	Cilnidipine	−15.04	−18.09	−20.50	−20.97
36	Naturetin	37.25	25.69	47.31	30.57
37	Alapuli	31.14	25.87	−5.07	−42.29
38	Candesartan Cilexetil	3.86	9.36	13.88	−15.36

**Table 5:** The calibration curve, R^2^, linear range, SDL, and LOQ of quality control compounds.

Index	Compounds	Calibration Curve	R^2^	Linear Range (mg/kg)	SDLs (mg/kg)	LOQs (mg/kg)
1	Clonidine	y = 1.357 × 10^4^ x + 5.645 × 10^3^	0.997	0.005–1.25	0.02	0.02
2	Tadalafil	y = 1.690 × 10^3^ x + 2.992 × 10^2^	0.998	0.05–1.25	0.06	0.06
3	Phenolphthalein	y = 3.082 × 10^3^ x + 1.863 × 10^3^	0.999	0.05–1.25	0.02	0.06
4	Glibenclamide	y = 5.736 × 10^3^ x + 6.537 × 10^2^	0.997	0.005–1.25	0.03	0.03
5	Nifedipine	y = 1.148 × 10^4^ x + 5.459 × 10^3^	0.999	0.050–1.25	0.02	0.06
6	Pseudovardenafil	y = 2.097 × 10^4^ x + 3.956 × 10^3^	0.999	0.005–1.25	0.01	0.01
7	Gliclazide	y = 1.415 × 10^4^ x + 2.926 × 10^3^	0.998	0.005–1.25	0.02	0.02
8	Norneosildenafil	y = 3.757 × 10^3^ x + 3.676 × 10^3^	0.998	0.250–5.00	0.25	0.25
9	Sildenafil	y = 1.201 × 10^4^ x + 1.425 × 10^3^	0.998	0.005–1.25	0.01	0.01
10	Acetildenafil	y = 1.904 × 10^4^ x + 6.133 × 10^3^	0.999	0.005–1.25	0.01	0.01
11	Dimethylacetildenafi	y = 2.807 × 10^4^ x + 1.151 × 10^4^	0.998	0.005–1.25	0.03	0.03
12	Acetylvardenafil	y = 3.075 × 10^4^ x + 4.740 × 10^3^	0.999	0.005–1.25	0.03	0.03
13	Bupropion	y = 9.165 × 10^3^ x + 5.981 × 10^2^	0.998	0.050–1.25	0.06	0.06
14	Phenformin	y = 1.563 × 10^4^ x + 7.821 × 10^3^	0.998	0.005–1.25	0.02	0.02
15	Hydroxyhomosildenafil	y = 6.718 × 10^3^ x + 6.591 × 10^2^	0.997	0.005–1.25	0.01	0.01
16	Atenolol	y = 7.130 × 10^3^ x + 1.404 × 10^3^	0.998	0.05–1.250	0.06	0.06
17	Prazosin	y = 4.231 × 10^4^ x + 1.204 × 10^4^	0.998	0.005–1.25	0.02	0.02
18	Reserpine	y = 7.438 × 10^3^ x ± 4.193 × 10^3^	0.997	0.050–1.25	0.1	0.1
19	Rimonabant	y = 1.167 × 10^4^ x + 2.931 × 10^3^	0.998	0.005–1.25	0.03	0.03
20	Tolazamide	y = 1.196 × 10^4^ x + 4.262 × 10^3^	0.998	0.005–1.25	0.02	0.02
21	Glipizide	y = 4.080 × 10^3^ x + 3.622 × 10^2^	0.998	0.050–1.25	0.03	0.06
22	Orlistat	y = 5.185 × 10^4^ x + 1.409 × 10^5^	0.999	0.250–5.00	0.25	0.25
23	Benfluorex	y = 3.164 × 10^4^ x + 6.754 × 10^3^	0.998	0.005–1.25	0.01	0.01
24	Diazepam	y = 1.555 × 10^4^ x + 1.074 × 10^4^	0.998	0.005–1.25	0.01	0.01
25	Noracetildenafil	y = 1.957 × 10^4^ x + 8.716 × 10^4^	0.999	0.250–5.00	0.5	0.5
26	Alprazolam	y = 1.984 × 10^4^ x + 8.650 × 10^3^	0.997	0.005–1.25	0.01	0.01
27	Triazolam	y = 1.544 × 10^4^ x + 6.287 × 10^3^	0.998	0.005–1.25	0.01	0.01
28	Nitrazepam	y = 2.273 × 10^3^ x + 1.218 × 10^3^	0.998	0.050–1.25	0.06	0.06
29	Lorazepam	y = 1.900 × 10^3^ x ± 9.330 × 10^1^	0.997	0.050–1.25	0.06	0.06
30	Zolpidem	y = 7.147 × 10^4^ x + 2.057 × 10^4^	0.997	0.005–1.25	0.003	0.003
31	Homosildenafil	y = 1.028 × 10^4^ x + 3.776 × 10^3^	0.997	0.005–1.25	0.02	0.02
32	Estazolam	y = 6.358 × 10^3^ x + 1.294 × 10^3^	0.998	0.005–1.25	0.03	0.03
33	Secobarbital	y = 3.220 × 10^3^ x + 1.158 × 10^3^	0.999	0.250–5.00	0.5	0.5
34	Amobarbital	y = 2.826 × 10^3^ x + 9.493 × 10^3^	0.998	0.250–5.00	0.5	0.5
35	Cilnidipine	y = 4.089 × 10^4^ x + 3.133 × 10^3^	0.998	0.005–1.25	0.03	0.03
36	Naturetin	y = 5.955 × 10^4^ x + 2.971 × 10^4^	0.999	0.050–1.25	0.1	0.1
37	Alapuli	y = 6.232 × 10^3^ x + 4.108 × 10^2^	0.999	0.050–1.25	0.1	0.1
38	Candesartan Cilexetil	y = 2.044 × 10^4^ x + 2.265 × 10^2^	0.999	0.050–1.25	0.1	0.1

**Table 6:** The spiked recoveries and relative standard deviations of quality control compounds.

Compounds	Spiked Levels	Tablets		Liquid Beverages		Solid Beverages		Oral Liquids	
		Recoveries (%)	RSD (%)	Recoveries (%)	RSD (%)	Recoveries (%)	RSD (%)	Recoveries (%)	RSD (%)
Clonidine	0.1	92.11	2.89	91.36	1.73	80.11	3.23	95.3	6.13
	0.2	107.19	2.9	91.84	1.3	91.01	4.35	92.14	3.24
	1.0	96.21	3.74	87.97	3.74	84.5	2.24	90.7	5.4
Tadalafil	0.1	90.77	5.25	94.12	1.7	83.12	4.69	87.8	2.58
	0.2	108.33	3.42	97.91	2.74	89.54	4.54	93.41	5.67
	1.0	93.43	2.56	98.72	1.78	89.05	0.63	92.96	4.83
Phenolphthalein	0.1	92.45	3.44	93.21	4.57	88.27	4.11	93.24	3.69
	0.2	103.64	6.19	96.8	5.22	97.08	2.65	96.33	5.91
	1.0	95.31	4.11	92.22	2.93	89.21	1.53	95.92	4.03
Glibenclamide	0.1	81.5	5.4	85.86	3.75	81.73	4.84	82.52	4.16
	0.2	90.92	2.51	87.08	1.42	89.25	2.35	82.71	3.99
	1.0	80.96	2.94	84.55	2.09	86.59	0.31	80.1	0.74
Nifedipine	0.1	86.56	5.12	87.22	4.06	83.28	3.29	91.36	5.24
	0.2	100.85	2.79	89.61	2.34	94.45	5.75	98.12	1.7
	1.0	94.24	1.65	90.16	3.2	93.15	1.06	96.95	2.93
Pseudovardenafil	0.1	93.71	1.94	88.53	1.79	83.05	5.07	92.73	1.55
	0.2	106.12	1.41	91.84	2.92	93.78	5.16	97.26	4.92
	1.0	96.28	2.81	91.94	3.83	91.67	1	86.93	2.14
Gliclazide	0.1	80.14	4.75	81.2	2.45	80.31	2.67	80.36	3.51
	0.2	85.81	1.95	84.53	2.22	87.78	2.58	82.35	3.37
	1.0	80.65	4.64	85.22	4.23	81.17	1.05	80.52	2.73
Norneosildenafil	0.1	97.46	3.18	92.62	5.25	81.56	4.16	92.84	2.83
	0.2	110.21	1.6	94.69	2.4	98.44	2.38	94.4	2.71
	1.0	100.76	1.51	91.23	2.52	96.97	0.82	96.48	0.98
Sildenafil	0.1	92.14	2.93	90.28	1.05	81.97	1.95	89.93	1.92
	0.2	106.31	2.2	91.48	0.67	89.75	2.49	92.67	1.48
	1.0	96.18	1.99	88.94	3.1	86.13	1.85	91.17	3.77
Acetildenafil	0.1	87.5	2.71	92.01	0.97	82.24	4.19	86.45	2.83
	0.2	100.57	3.23	96.66	3.35	92.77	2.69	89.31	1.44
	1.0	92.51	1.02	96.58	4.36	89.92	1.52	85.72	2.4
Dimethylacetildenafi	0.1	93.87	2.91	91.25	3.45	102.16	4.44	102.4	3.93
	0.2	95.53	3.63	91.42	6.06	95.56	5.29	113.59	3.55
	1.0	90.49	5.55	91.43	2.62	97.57	1.56	100.59	2.7
Acetylvardenafil	0.1	98.14	2.5	88.4	2.68	97.64	4.75	101.83	2.82
	0.2	98.43	1.2	87.31	4.65	91.49	8.36	99.66	3.36
	1.0	93.87	2.91	92.74	1.97	95.88	3.24	91.89	2.27
Bupropion	0.1	92.55	3.74	95.84	4.8	80.89	5.13	94.77	3.55
	0.2	104.68	1.03	98.01	5.09	94.84	4.3	94.88	2.45
	1.0	92.35	6.19	95.77	1.68	89.6	3.56	86.76	3.91
Phenformin	0.1	90.83	2.61	80.4	4.04	80.63	5.61	98.2	4.2
	0.2	103.23	2.67	90.2	3.72	92.42	2.81	87.11	4.73
	1.0	95.93	1.9	85.38	3.58	84.17	0.54	80.4	2.77
Hydroxyhomosildenafil	0.1	94.64	2.05	90.59	2.18	87.69	4.19	98.3	2.75
	0.2	103.79	0.71	91.14	1.79	93.15	4.09	92.03	5.01
	1.0	95.28	2.34	89.57	3.2	90.73	0.64	91.54	1.57
Atenolol	0.1	90.36	5.1	95.32	4.85	81	4.31	94.93	2.64
	0.2	101.56	4.47	98.18	3.48	93.22	2.41	91.58	4.82
	1.0	85	3.95	90.41	2.69	89.04	1.72	86.21	5.48
Prazosin	0.1	89.65	1.32	92.4	2.47	80.27	3.17	85.61	2.22
	0.2	105.81	1.75	94.2	2.41	90.47	2.27	88.41	2.78
	1.0	92.98	4.31	91.05	3.28	90.09	5.42	87.06	2.22
Reserpine	0.1	94.09	5.11	86.96	5.24	84.2	5.11	92.15	3.52
	0.2	102.36	4.28	85.33	4.66	94.26	8.2	86.61	4.2
	1.0	93.08	2.09	91.58	2.08	91.52	1.29	82.03	1.9
Rimonabant	0.1	89.22	3.9	89.08	2.76	81.93	4.57	90.3	2.4
	0.2	104.41	2.81	89.77	4.01	96.5	5.23	94.14	2.96
	1.0	97.11	2.09	87.26	2.58	96.81	1.45	93.22	1.42
Tolazamide	0.1	81.85	1.86	86.32	4.32	80.43	3.64	82.01	2.58
	0.2	85.92	2.12	85.19	2.16	86.97	3.73	81.91	3.6
	1.0	80.03	3.56	80.92	3.97	84.79	0.82	80.17	3.42
Glipizide	0.1	80.67	4.17	84	3.64	80.2	4.65	87.75	3.41
	0.2	88.74	1.87	83.84	1.86	90.52	5.95	84.35	2.99
	1.0	83.65	4.79	82.66	2.08	87.81	0.48	80.38	2.38
Orlistat	0.1	91.83	5.47	85.87	4.26	92.57	4.97	89.93	5.69
	0.2	95.53	4.04	98.24	4.11	101.39	4.47	83.56	5.78
	1.0	86.51	3.11	90.5	4.07	95.64	2.95	87.32	2.74
Benfluorex	0.1	90.32	2.14	84.72	2.53	80.15	1.83	88.17	1.71
	0.2	102.91	1.34	87.14	2.1	90.16	3.42	89.33	3.31
	1.0	92.26	2.75	87.42	2.13	84.98	1.81	86.93	3.01
Diazepam	0.1	93.43	1.55	94.16	2.17	80.04	3.29	95.93	1.39
	0.2	109.56	1.4	98.74	1.83	93.73	2.55	99.25	2.47
	1.0	98.44	2.94	93	2.37	90.01	1.53	92.64	1.9
Noracetildenafil	0.1	83.05	0.84	94.85	3.1	81.78	5.71	88.15	2.24
	0.2	96.7	2.31	96.07	3.04	92.47	3.03	92.05	1.5
	1.0	87.1	1.9	90.08	2.7	86.22	2.21	84.12	4.6
Alprazolam	0.1	90.49	1.46	95.13	2.67	86.79	2.43	92.98	2.67
	0.2	107.34	2.28	99.3	2.11	100.91	1.82	92.19	3.48
	1.0	98.93	2.77	97	3.07	98.86	0.23	90.37	2.44
Triazolam	0.1	91.3	2.73	95.48	3.26	87.17	3.14	93.6	2.62
	0.2	107.55	2.59	98.56	2.2	102.08	1.48	93.15	4.43
	1.0	101.16	1.45	96.52	2.53	97.59	1.17	91.7	2.53
Nitrazepam	0.1	88.25	4.34	92.55	2.11	86.13	7.49	87.65	2.37
	0.2	105.96	1.32	93.19	2.18	91.52	3.52	93.24	4.8
	1.0	96.73	2.69	90.66	2.41	89.31	2.18	94.2	2.22
Lorazepam	0.1	91.64	3.6	90.38	4.22	83.99	5.07	85.67	9.37
	0.2	107.95	4.06	92.3	5.31	95.12	4.16	88.86	5.87
	1.0	97.51	3.45	92.03	3.22	96.91	1.32	95	3.01
Zolpidem	0.1	94.97	2.36	88.73	2.21	81.91	4.15	91.97	2.51
	0.2	110.93	2.86	91.26	4.14	97.98	4.54	96.78	2.91
	1.0	100.66	3.59	87.44	3.75	90.58	2.2	96.92	4.5
Homosildenafil	0.1	89.22	2.81	90.78	2.27	85.53	2.47	90.49	1.44
	0.2	106.66	1.07	91.00	2.29	93.15	3.71	90.82	3.39
	1.0	99.39	1.94	88.00	1.8	89.89	2.15	85.45	3.19
Estazolam	0.1	87.69	4.02	93.26	4.35	84.09	4.85	94.13	4.64
	0.2	107.29	2.49	95.05	3.38	97.07	2.68	100.29	4.9
	1.0	95.95	1.46	91.61	3.89	103.67	0.89	88.99	2.62
Secobarbital	0.1	102.47	3.42	91.62	2.76	82.47	2.51	85.49	3.44
	0.2	109.21	4.29	99.46	2.62	95.54	4.91	84.71	4.71
	1.0	95.7	2.97	101.88	2.88	89.24	5.60	82.07	3.96
Amobarbital	0.1	98.4	3.71	98.51	3.44	81.64	4.77	92.36	5.24
	0.2	108.89	5.23	102.5	5.83	100.48	3.86	87.75	4.83
	1.0	97.76	3.47	100.21	0.95	93.37	5.14	81.23	3.62
Cilnidipine	0.1	79.83	4.49	78.10	3.93	82.39	4.01	81.06	3.73
	0.2	79.38	7.66	86.36	3.96	92.81	3.34	91.45	3.08
	1.0	75.62	7.36	85.88	1.74	89.43	5.39	93.16	8.26
Naturetin	0.1	82.91	6.52	81.89	5.75	83.62	2.24	83.95	2.90
	0.2	90.63	4.11	92.37	2.32	91.91	4.30	89.63	5.11
	1.0	90.90	3.22	84.61	5.31	84.44	1.50	85.45	2.51
Alapuli	0.1	70.82	4.50	78.98	5.03	77.58	2.97	74.41	6.55
	0.2	71.32	2.95	78.45	6.30	79.61	6.15	79.85	4.32
	1.0	74.30	1.83	79.95	2.19	78.48	3.81	73.79	3.08
Candesartan Cilexetil	0.1	70.09	5.83	69.76	4.60	82.69	3.73	67.97	7.48
	0.2	72.95	6.10	78.25	3.02	88.84	2.38	70.18	5.85
	1.0	80.32	5.01	81.17	3.20	84.44	2.61	71.67	6.96

## Data Availability

The original contributions presented in the study are included in the article/Supplementary Materials. Further inquiries can be directed to the corresponding author.

## Supplementary Materials

The following supporting information can be downloaded at https://www.mdpi.com/article/10.3390/foods15112001/s1: Figure S1: Separation chromatogram of isomers under different mobile phase conditions. Figure S2: Chromatograms of the 38 quality control compounds acquired in ESI+ and ESI− modes. Figure S3: Comparison of solvent extraction effects for representative compounds. Figure S4: Comparison of extraction methods and effects for representative compounds. Figure S5: Comparison of extraction time effects for representative compounds. Figure S6: Comparison of purification effects of representative compounds. Figure S7: Comparison of PSA dosage effects for representative compounds. Figure S8: Comparison of C18 dosage effects for representative compounds. Table S1: logP, pKa, and category information of the 38 quality control compounds.

## Abbreviations

The following abbreviations are used in this manuscript:

DSPE	dispersive solid-phase extraction
UPLC-Q-TOF-MS	ultra-performance liquid chromatography–quadrupole-time-of-flight mass spectrometry
RSDs	relative standard deviations
SDLs	screening detection limits
LOQs	limits of quantifications
LC-MS/MS	liquid chromatography–triple-quadrupole mass spectrometry
MRM	multiple-reaction monitoring
HRMS	high-resolution mass spectrometry
IDA	information-dependent acquisition
S/N	signal-to-noise ratio
XIC	extract ion chromatogram
ME	matrix Effect
R^2^	linear correlation coefficient
DBS	dynamic background subtraction
MS	precursor exact mass
MS/MS	fragment ion mass
