# Supplementary material for: Suspected and Non-Targeted Screening of Non-Edible Substances in Food by UPLC-Q-TOF-MS
Source: Foods. 2026 Jun 3;15(11):2001. doi: 10.3390/foods15112001 (PMC13257398; doi:10.3390/foods15112001)
Supplement: Supplementary file 1 [file foods-15-02001-s001.zip › foods-4200567-supplementary.pdf]

Supplementary Materials

The following supplementary materials are available online:
Figure S1: Separation chromatogram of isomers under different mobile phase conditions.
Figure S2: Chromatograms of the 38 quality control compounds acquired in ESI+ and ESI− modes.
Figure S3: Comparison of solvent extraction effects for representative compounds.
Figure S4: Comparison of extraction methods and effects for representative compounds.
Figure S5: Comparison of extraction time effects for representative compounds.
Figure S6: Comparison of purification effects of representative compounds.
Figure S7: Comparison of PSA dosage effects for representative compounds.
Figure S8: Comparison of C18 dosage effects for representative compounds.
Table S1: logP, pKa, and category information of the 38 quality control compounds.

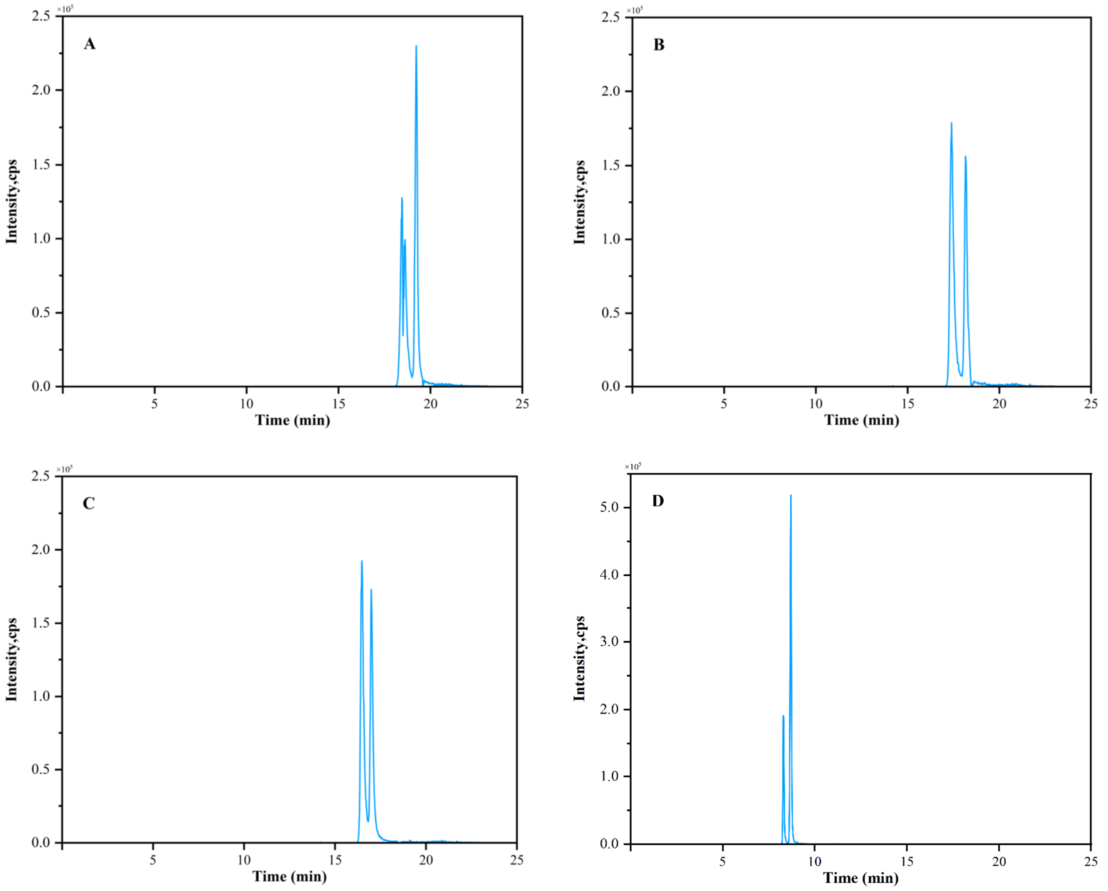

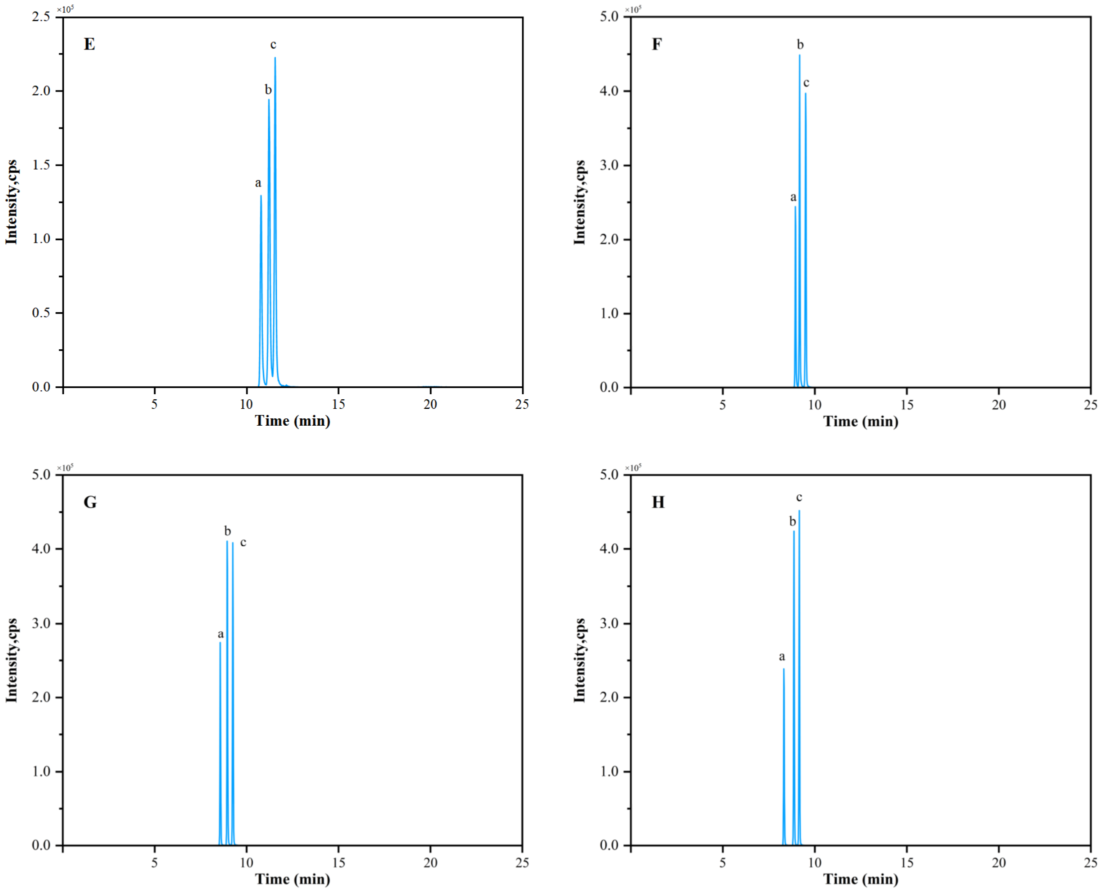

Figure S1. Separation chromatogram of isomers.A:2.5mmol/L ammonium formate aqueous solution-methanol; B:5.0mmol/L ammonium formate aqueous solution-methanol; C:10.0 mmol/L ammonium formate aqueous solution-methanol; D:5.0 mmol / L ammonium formate aqueous solution-acetonitrile; E:5.0 mmol /L ammonium formate aqueous solution and methanol-acetonitrile (1:1,v/v); F:5.0 mmol/L ammonium formate aqueous solution(containing 0.01% formic acid) and methanol-acetonitrile (1:1,v/v); G:5.0 mmol/L ammonium formate aqueous solution(containing 0.05% formic acid) and methanol-acetonitrile (1:1,v/v); H:5.0 mmol/L ammonium formate aqueous solution (containing 0.1% formic acid) and methanol-acetonitrile (1:1,v/v). a is acetildenafil, b is dimethyl acetildenafil, and c is acetylvardenafil.

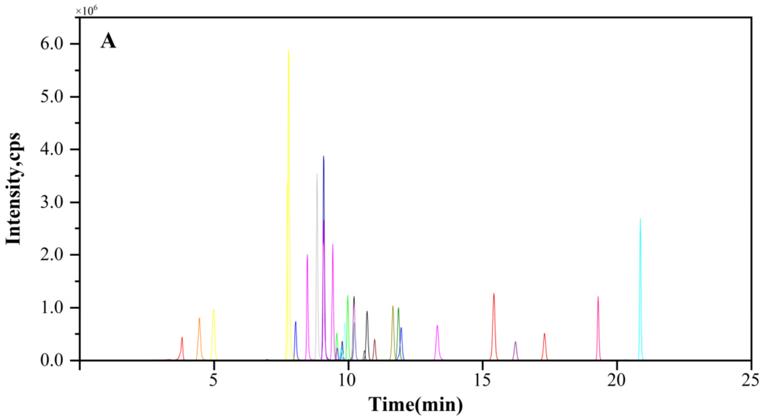

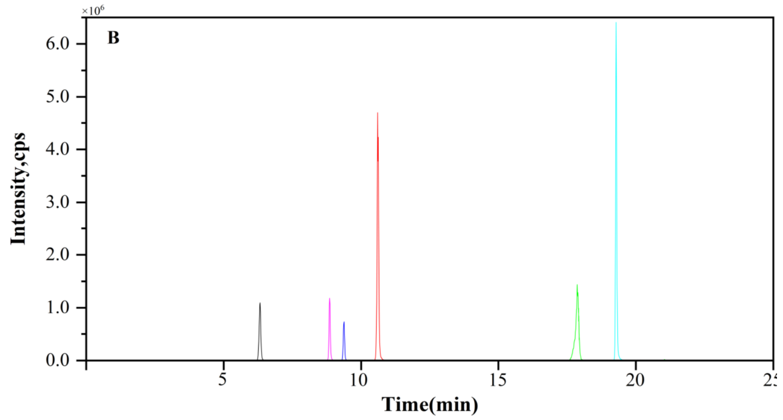

Figure S2. Chromatograms of the 38 quality control compounds acquired in ESI+(A) and ESI-(B)

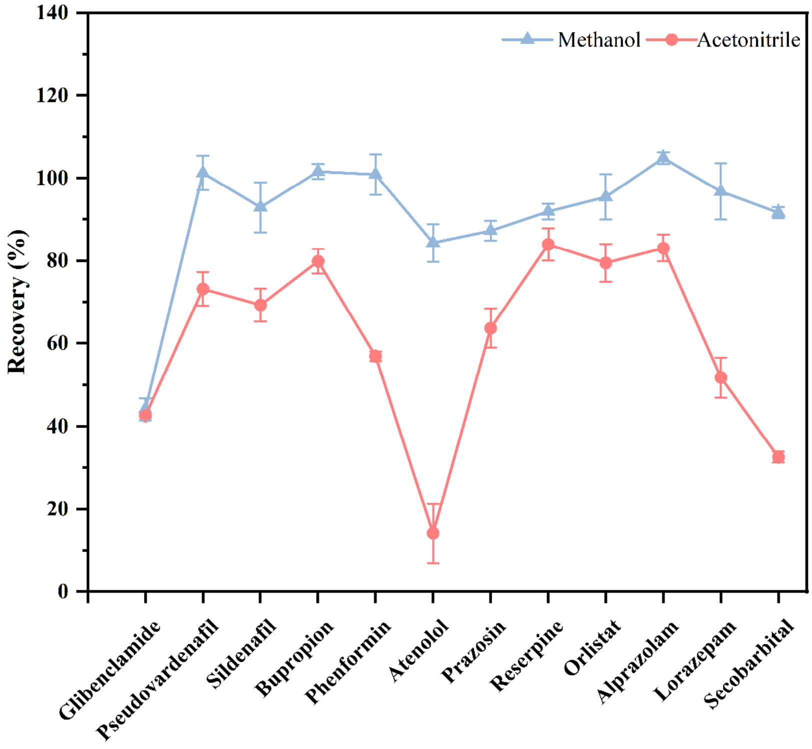

Figure S3. Comparison of solvent extraction effects for representative compounds.

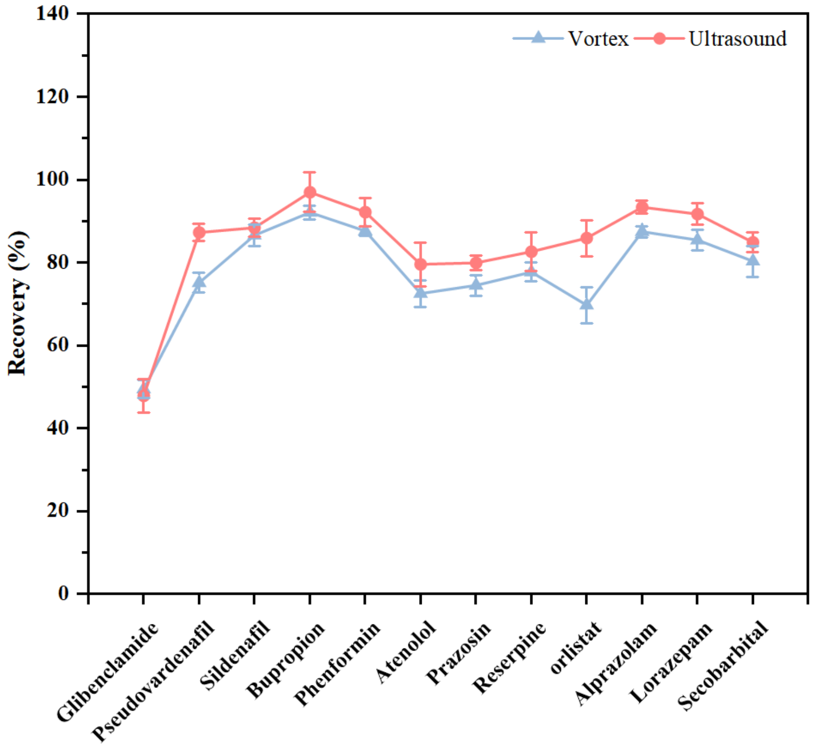

Figure S4. Comparison of extraction methods and effects of representative compounds.

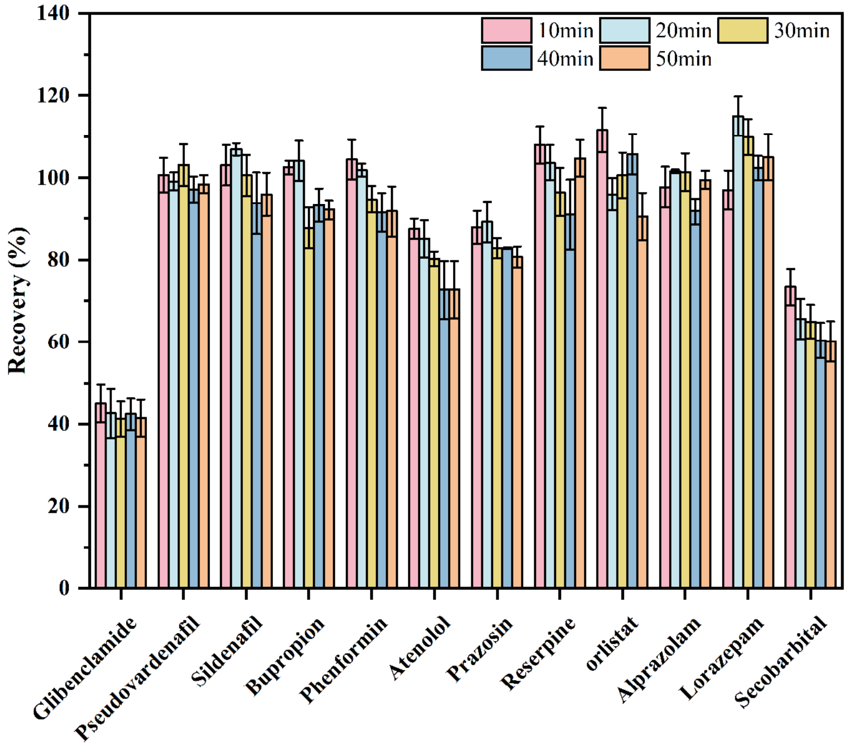

Figure S5. Comparison of extraction time effects of representative compounds.

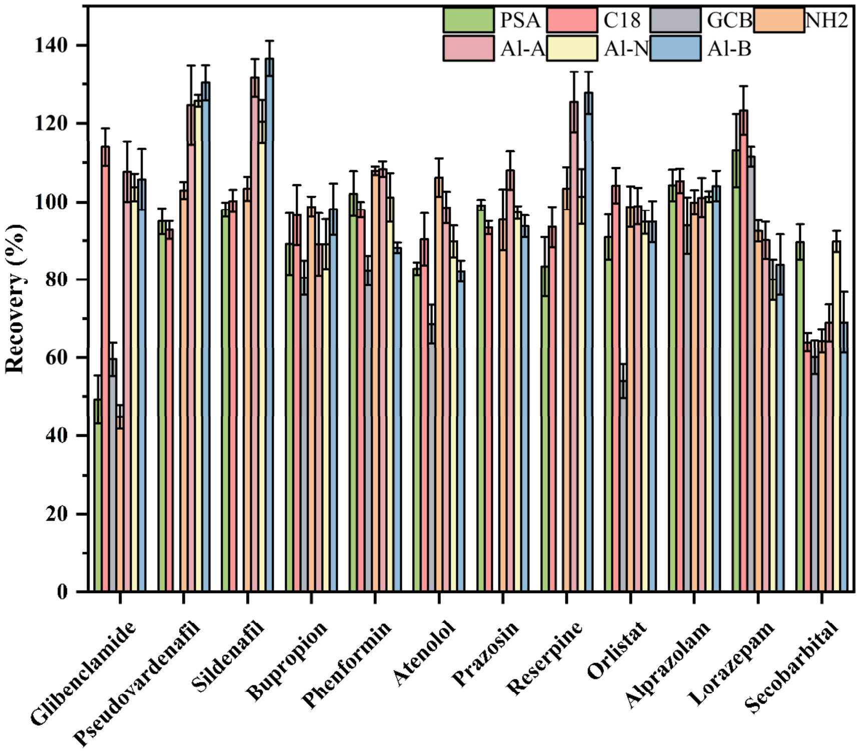

Figure S6. Comparison of purification effects of representative compounds.

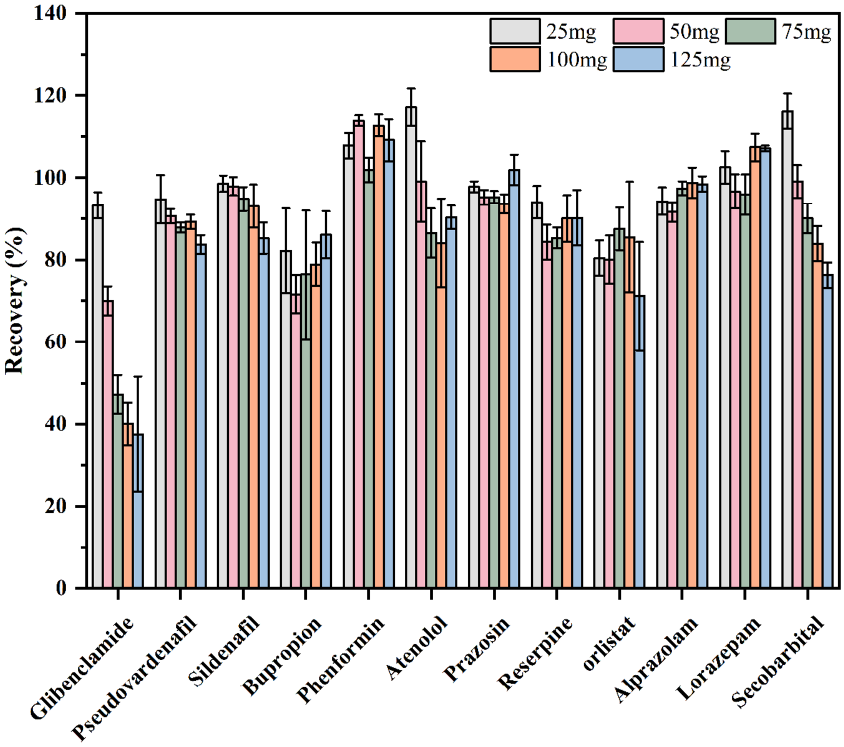

Figure S7. Comparison of purification effects of representative compounds PSA dosage.

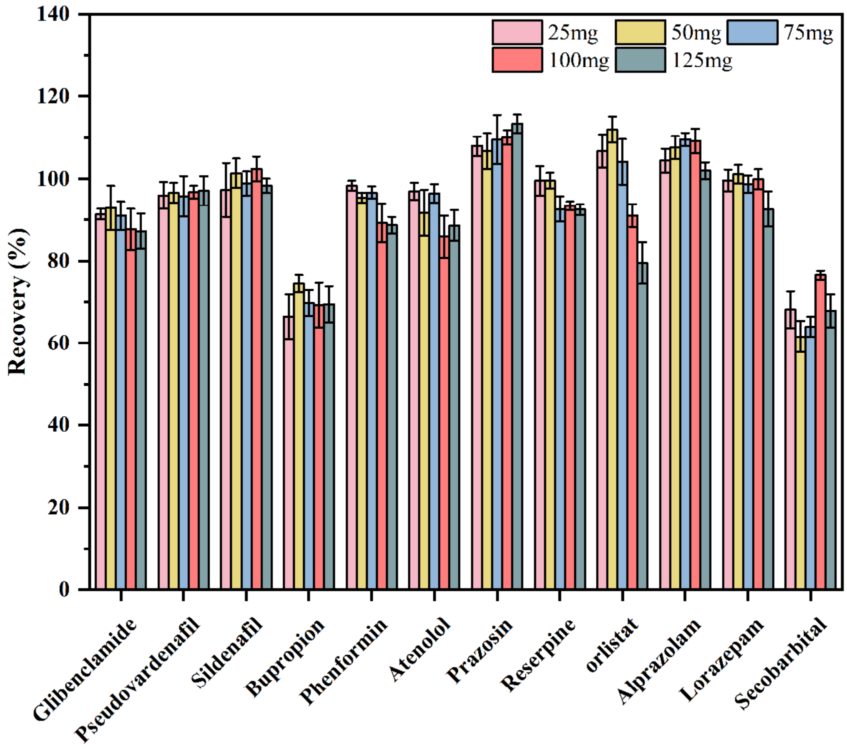

Figure S8. Comparison of purification effects of representative compounds C_{18} dosage.

Table S1 logP,and category information of quality control compounds

Compound Name	Formula	logP	Category
Tadalafil	$C_{22}H_{19}N_3O_4$	1.72	Antifatigue agents
Pseudovardenafil	$C_{22}H_{29}N_5O_4S$	2.84	Antifatigue agents
Norneosildenafil	$C_{22}H_{29}N_5O_4S$	2.56	Antifatigue agents
Sildenafil	$C_{22}H_{30}N_6O_4S$	2.13	Antifatigue agents
Acetildenafil	$C_{25}H_{34}N_6O_3$	3.45	Antifatigue agents
Dimethyl acetildenafil	$C_{25}H_{34}N_6O_3$	3.78	Antifatigue agents
Acetylvardenafil	$C_{25}H_{34}N_6O_3$	3.52	Antifatigue agents
Hydroxyhomosildenafil	$C_{23}H_{32}N_6O_5S$	2.15	Antifatigue agents
Homosildenafil	$C_{23}H_{32}N_6O_4S$	2.98	Antifatigue agents
Noracetildenafil	$C_{24}H_{32}N_6O_3$	3.12	Antifatigue agents
Alprazolam	$C_{17}H_{13}ClN_4$	2.92	Sedative-hypnotics
Triazolam	$C_{17}H_{12}Cl_2N_4$	3.15	Sedative-hypnotics

Estazolam	$C_{16}H_{11}ClN_4$	2.86	Sedative-hypnotics
Diazepam	$C_{16}H_{13}ClN_2O$	2.82	Sedative-hypnotics
Nitrazepam	$C_{15}H_{11}N_3O_3$	2.16	Sedative-hypnotics
Lorazepam	$C_{15}H_{10}Cl_2N_2O_2$	2.39	Sedative-hypnotics
Zolpidem	$C_{19}H_{21}N_3O$	2.83	Sedative-hypnotics
Amobarbital	$C_{11}H_{18}N_2O_3$	1.78	Sedative-hypnotics
Secobarbital	$C_{12}H_{18}N_2O_3$	1.9	Sedative-hypnotics
Glibenclamide	$C_{23}H_{28}ClN_3O_5S$	4.76	Hypoglycemic agents
Gliclazide	$C_{15}H_{21}N_3O_3S$	2.12	Hypoglycemic agents
Phenformin	$C_{10}H_{15}N_5$	0.32	Hypoglycemic agents
Tolazamide	$C_{14}H_{21}N_3O_3S$	2.24	Hypoglycemic agents
Glipizide	$C_{21}H_{27}N_5O_4S$	2.68	Hypoglycemic agents
Clonidine	$C_9H_9Cl_2N_3$	1.59	Antihypertensive agents
Nifedipine	$C_{17}H_{18}N_2O_6$	2.2	Antihypertensive agents
Atenolol	$C_{14}H_{22}N_2O_3$	–0.16	Antihypertensive agents
Prazosin	$C_{19}H_{21}N_5O_4$	1.48	Antihypertensive agents
Reserpine	$C_{33}H_{40}N_2O_9$	3.02	Antihypertensive agents
Cilnidipine	$C_{27}H_{28}N_2O_7$	5.7	Antihypertensive agents
Naturetin	$C_{15}H_{14}F_3N_3O_4S_2$	2.76	Antihypertensive agents
Alapuli	$C_{20}H_{26}N_2O_5S$	3.18	Antihypertensive agents
Candesartan Cilexetil	$C_{33}H_{34}N_6O_6$	6.11	Antihypertensive agents
Phenolphthalein	$C_{20}H_{14}O_4$	2.41	Weight-loss compounds
Rimonabant	$C_{22}H_{21}Cl_3N_4O$	5.32	Weight-loss compounds
Orlistat	$C_{29}H_{53}NO_5$	7.12	Weight-loss compounds
Benfluorex	$C_{19}H_{20}F_3NO_2$	4.56	Weight-loss compounds
Bupropion	$C_{13}H_{18}ClNO$	3.12	Antidepressants
